# LRPPRC promotes glycolysis by stabilising LDHA mRNA and its knockdown plus glutamine inhibitor induces synthetic lethality via m^6^A modification in triple‐negative breast cancer

**DOI:** 10.1002/ctm2.1583

**Published:** 2024-02-19

**Authors:** Yuanhang Yu, Huifang Deng, Wenwen Wang, Shihan Xiao, Renjing Zheng, Lianqiu Lv, Han Wang, Jianying Chen, Bo Zhang

**Affiliations:** ^1^ Department of Breast and Thyroid Surgery Union Hospital, Tongji Medical College, Huazhong University of Science and Technology Wuhan China; ^2^ Cancer Center, Union Hospital, Tongji Medical College, Huazhong University of Science and Technology Wuhan China; ^3^ Department of Obstetrics and Gynecology Union Hospital, Tongji Medical College Huazhong University of Science and Technology Wuhan China; ^4^ Department of Gastrointestinal Surgery Union Hospital, Tongji Medical College, Huazhong University of Science and Technology Wuhan China

**Keywords:** LDHA, LRPPRC, metabolic reprogramming, N^6^‐methyladenosine, synthetic lethal, triple‐negative breast cancer

## Abstract

**Background:**

Targeted therapy for triple‐negative breast cancer (TNBC) remains a challenge. N6‐methyladenosine (m^6^A) is the most abundant internal mRNA modification in eukaryotes, and it regulates the homeostasis and function of modified RNA transcripts in cancer. However, the role of leucine‐rich pentatricopeptide repeat containing protein (LRPPRC) as an m^6^A reader in TNBC remains poorly understood.

**Methods:**

Western blotting, reverse transcription‐polymerase chain reaction (RT‐qPCR) and immunohistochemistry were used to investigate LRPPRC expression levels. Dot blotting and colorimetric enzyme linked immunosorbent assay (ELISA) were employed to detect m^6^A levels. In vitro functional assays and in vivo xenograft mouse model were utilised to examine the role of LRPPRC in TNBC progression. Liquid chromatography–mass spectrometry/mass spectrometry and Seahorse assays were conducted to verify the effect of LRPPRC on glycolysis. MeRIP‐sequencing, RNA‐sequencing, MeRIP assays, RNA immunoprecipitation assays, RNA pull‐down assays and RNA stability assays were used to identify the target genes of LRPPRC. Patient‐derived xenografts and organoids were employed to substantiate the synthetic lethality induced by LRPPRC knockdown plus glutaminase inhibition.

**Results:**

The expressions of LRPPRC and m^6^A RNA were elevated in TNBC, and the m^6^A modification site could be recognised by LRPPRC. LRPPRC promoted the proliferation, metastasis and glycolysis of TNBC cells both in vivo and in vitro. We identified lactate dehydrogenase A (LDHA) as a novel direct target of LRPPRC, which recognised the m^6^A site of LDHA mRNA and enhanced the stability of LDHA mRNA to promote glycolysis. Furthermore, while LRPPRC knockdown reduced glycolysis, glutaminolysis was enhanced. Moreover, the effect of LRPPRC on WD40 repeat domain‐containing protein 76 (WDR76) mRNA stability was impaired in an m^6^A‐dependent manner. Then, LRPPRC knockdown plus a glutaminase inhibition led to synthetic lethality.

**Conclusions:**

Our study demonstrated that LRPPRC promoted TNBC progression by regulating metabolic reprogramming via m^6^A modification. These characteristics shed light on the novel combination targeted therapy strategies to combat TNBC.

## Graphical abstract

1. LRPPRC is overexpressed in TNBC, enhancing glycolysis and promoting tumour progression.

2. LRPPRC promotes the stability of LDHA and WDR76 mRNA in an m^6^A‐dependent manner. LRPPRC knockdown decreases LDHA expression, attenuates glycolysis, simultaneously decreases WDR76 expression, and enhances glutaminolysis and energy replenishment.

3. LRPPRC knockdown, in conjunction with glutaminase inhibition, led to synthetic lethality in TNBC.

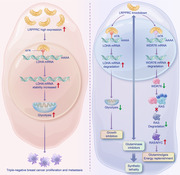

## 1 INTRODUCTION

Triple‐negative breast cancer (TNBC) represents the most aggressive histological subtype of breast cancer, and is characterised by an absence of estrogen receptor (ER), progesterone receptor (PR) and HER2 expression.^1^ While TNBC accounts for a relatively small proportion of breast cancer cases, it is culpable for a disproportionately higher mortality.^2^ Over the past decades, TNBC has been genetically, epigenetically and transcriptomically well‐studied, and was demonstrated to be driven by the epigenetic regulation of genes implicated in cell proliferation, survival and differentiation.^3^ Nonetheless, little is known about the exact molecular mechanisms by which epigenetic alterations are regulated and TNBCs are driven.

Post‐transcriptional modifications have gained the attention of researchers.^4^, ^5^, ^6^ Among 163 well‐established RNA modifications, N6‐methyladenosine (m^6^A) modification is the most common internal mRNA modification in eukaryotes.^7^ m^6^A modifications can be dynamically regulated. The modified segments, which were deposited by m^6^A methyltransferases (writers), removed by m^6^A demethylases (erasers) and recognised by m^6^A‐binding proteins (readers), dictate the fate of mRNA by modulating their splicing, translation or stability.^8^ Importantly, mounting evidence has shown that m^6^A readers play critical roles in the regulation of gene expression during breast cancer progression, serve various functions in different cellular regions and bind to m^6^A‐modified RNAs.^9^, ^10^, ^11^ Leucine‐rich pentatricopeptide repeat containing protein (LRPPRC), an m^6^A reader protein, was recently identified. However, its biological significance and mechanisms in the progression of TNBC are still unclear.

Reprogramming of energy metabolism is a hallmark of cancer.^12^ The aberrant catabolism of glucose and glutamine is considered to be two distinct features of cancer metabolism. A substantially increased consumption of glucose by tumours, compared to non‐proliferating normal tissues, was first described by Otto Warburg and is also known as the ‘Warburg effect’ or aerobic glycolysis.^13^ Aerobic glycolysis is characterised by increased glucose uptake and preferential lactate production, even in the presence of oxygen, and can support energy production and biosynthesis to promote proliferation and metastasis.^14^ Glutamine is the second principal growth‐supporting substrate and is required for the proliferation of tumourous cells.^13^ Emerging evidence has also demonstrated that novel tumour drivers modulate tumourigenesis and progression by regulating glycolysis and glutamine metabolism in TNBC. However, the role and mechanism of RNA modification, including m^6^A modification, in cancer metabolism remains elusive.

Previous studies have demonstrated that LRPPRC, a member of the pentatricopeptide repeat (PPR) family, is an RNA‐binding protein that regulates cellular growth, invasion, apoptosis and drug resistance, is highly expressed in various tumours and is associated with unfavourable prognosis.^15^ LRPPRC is mainly located in the mitochondria, cytoskeleton, endoplasmic reticulum and the inner and outer nuclear membrane. As a multifunctional protein, LRPPRC regulates a wide array of biological processes, including energy metabolism, stability and mRNA maturation. More importantly, Arguello et al. found that LRPPRC can chemically bind to m^6^A RNA by a proteomic approach.^16^ To date, no study has specifically explored the roles of LRPPRC in the progression of TNBC. Therefore, in this study, we investigated the possible role of LRPPRC in the progression of TNBC from the perspective of the m^6^A modification.

Our results showed that LRPPRC expression and m^6^A modification levels were aberrantly upregulated in TNBC, and that LRPPRC could recognise m^6^A modification sites in TNBC. Multi‐omic analysis revealed that elevated LRPPRC promoted glycolysis, growth and aggressiveness in TNBC cells by maintaining the stability of lactate dehydrogenase A (LDHA) mRNA through the m^6^A pathway. More importantly, LRPPRC knockdown attenuated glycolysis and inhibited tumour growth while increasing the cellular consumption of glutamine in TNBC. These effects could be ascribed to the decreased WD40 repeat domain‐containing protein 76 (WDR76) mRNA stability resulting from LRPPRC knockdown through m^6^A modification. As a consequence, the lowered WDR76 mRNA stability led to downregulated WDR76 expression, and further decreased RAS degradation via WDR76 ubiquitination, elevated RAS and MYC expression, and thereby promoted glutaminolysis. Consequently, LRPPRC knockdown, in conjunction with glutaminase inhibition, led to synthetic lethality in TNBC. Overall, our data demonstrated that the m^6^A reader LRPPRC played a critical oncogenic role in TNBC progression, and the LRPPRC/LDHA/WDR76 axis might serve as an important therapeutic target for metabolic reprogramming in TNBC.

## 2 METHODS

### 2.1 TNBC tissue specimens and cell lines

TNBC and adjacent normal breast tissues were obtained from 18 patients who had undergone radical mastectomy at the Department of Breast and Thyroid Surgery, Wuhan Union Hospital, Huazhong University of Science and Technology, Wuhan, China. The cell lines MCF10A and MDA‐MB‐468 were obtained from Guangzhou Cellcook Biotech, and MCF‐7, T47D, BT474, SKBR3, MDA‐MB‐231, MDA‐MB‐453, BT549, BT20 and HCC1937 were obtained from the American Type Culture Collection. These cell lines were cultured in recommended media and were validated to be free of Mycoplasma. These cells were subjected to short tandem repeat analysis.

### 2.2 Plasmids, siRNA transfection and recombinant lentivirus

shLRPPRCs‐knocked down plasmids, OE‐LRPPRC‐overexpressed plasmids, OE‐LDHA‐overexpressed plasmids and the control (empty vector) were obtained from GENECHEM. To stably knocked down LRPPRC, the hU6‐MCS‐CBh‐gcGFP‐IRES‐puromycin vector was used. To stably overexpress LRPPRC (NM_133259), the Ubi‐MCS‐3FLAG‐SV40‐EGFP‐IRES‐puromycin vector was employed. LDHA expression plasmid was constructed by cloning LDHA (NM_001165414) gene into CMV‐MCS‐SV40‐neomycin vector. The shRNA/overexpression vectors of LRPPRC were introduced into cancer cell lines by lentiviral infection, and recombinant lentiviral particles were produced by GENECHEM. The sequence of si‐LRPPRC was synthesised from sh‐LRPPRC and purchased from Tsingke. The sequences of shRNAs are listed in Table S1. Forty‐eight hours after infection, puromycin (5 μg/mL) was added to the culture medium for selection, and LRPPRC expression was detected by qRT‐PCR or immunoblotting.

### 2.3 RNA extraction and qRT‐PCR

Total RNA was extracted from tissues and cells by using RNAiso Reagent (Takara) according to the user's manual. For qRT‐PCR, RNA was reversely transcribed to cDNA by employing the PrimeScript RT reagent kit (Takara RR037A). The levels of RNA transcripts were analysed on the StepOne plus PCR system (ABI) by utilising the TB Green Premix Ex Taq II kit (Takara RR820A). All samples were normalised to GAPDH. Primers are listed in Table S2.

### 2.4 Western blot assays

Western blotting was carried out as described previously. In these experiments, antibodies against LRPPRC (Proteintech, 21175‐1‐AP), LDHA (Cell Signaling Technology, 3582), WDR76 (Proteintech, 25528‐1‐AP), MYC (Santa Cruz Biotechnology, sc‐40), HuR (Proteintech, 11910‐1‐AP) and PABPC1 (Proteintech, 10970‐1‐AP) were used.

### 2.5 Immunofluorescence

Cells were seeded into confocal dish and fixed in 4% paraformaldehyde for 30 min, permeabilised in .1% Triton X‐100 for 10 min and probed with an anti‐LRPPRC (1:100, Santa Cruz Biotechnology, sc‐166178) and anti‐m^6^A antibody (1:100, ABclonal, A19841). Nuclei were detected with 4′,6‐diamidino‐2‐phenylindole (Invitrogen). The specific procedures were performed as described previously.

### 2.6 Immunohistochemical staining

Immunohistochemical (IHC) or haematoxylin–eosin staining of TNBC tissues and xenografts used were performed as previously reported. The primary antibodies were as follows: anti‐LRPPRC (1:200, GeneTex, GTX109558), anti‐LDHA (1:200, ABclonal, A1146), anti‐WDR76 (1:200, Proteintech, 25528‐1‐AP) and anti‐Ki‐67 (1:200, Proteintech, 27309‐1‐AP) antibodies. The IHC score was calculated by multiplying the positive rate by the intensity score.

### 2.7 Cell proliferation assays

Cell proliferation was assessed by employing cell counting kit‐8 (CCK‐8) assay, 5‐ethynyl‐2′‐deoxyuridine (EdU) assay and colony formation assay. For CCK‐8 assay, cells (5 × 10^3^ cells/well) were seeded into 96‐well plates. CCK‐8 solution (Selleck) was added at the indicated time points, and absorbance was measured at 450 nm on a microplate reader. For EdU and colony formation assays, cells were seeded into 96‐well plates (at 2 × 10^4^) and six‐well plates (at 1 × 10^3^) cells, respectively.

### 2.8 Transwell and wound healing assays

For the cell migration and invasion transwell assays, the 24‐well transwell chamber system was used. Cells (3 × 10^4^) in 200 μL serum‐free media were seeded into the top chambers of transwell inserts, while 700 μL culture medium with 20% foetal bovine serum was added to the lower chamber. The upper 24‐well transwells were coated with Matrigel (Sigma–Aldrich) before plating cells during invasion assays. The cells were stained with crystal violet, imaged and counted under a microscope (×20). For wound healing assay, cells (5 × 10^5^) were cultured in six‐well plates, with 2 mL for each well. Once the cells were overgrown, they were scratched in a straight line with a pipette tip and exfoliated cells were removed. At the indicated time points, the distance of cell migration to the middle of the scratch was observed under a light microscope with green colour, and the cells were imaged at 40× magnifications.

### 2.9 Apoptosis assay

Cells were stained with AnnexinV‐PE/7‐AAD (BD, 559763) for 30 min at 4°C according to the manufacturer's instructions and apoptosis was analysed on a BD FACS CantoII Flow cytometer (BD Biosciences).

### 2.10 Co‐immunoprecipitation

Co‐immunoprecipitation (Co‐IP) assay was conducted by using the Co‐Immunoprecipitation Kit (Beyotime Biotechnology, P2197M) according to the manufacturer's protocol. Cells were harvested with the immunoprecipitation (IP) buffer on ice. After centrifugation at 12 000 rpm for 15 min, the supernatant was harvested. Anti‐m^6^A antibody (5 μg, Abcam, ab151230) was bound to protein A + G agarose gel and was shaken for 1 h at room temperature. It was then added to the sample and incubated overnight at 4°C. Then, denatured immunoprecipitated samples were added to Sodium dodecyl sulfate‐Polyacrylamide gel electrophoresis (SDS–PAGE) gels and transferred onto Polyvinylidene fluoride (PVDF) membranes, followed by immunoblotting with the corresponding antibodies.

### 2.11 Quantification of mRNA m^6^A

The mRNA m^6^A was quantitatively evaluated by the m^6^A dot‐blot assay and the colorimetrically quantified by m^6^A RNA methylation assay. For RNA m^6^A dot‐blot assay, intact mRNA was purified from total RNA by using the Oligotex Direct mRNA Mini Kit (QIAGEN, 72022). The concentration of purified mRNA was spectrophotometrically determined by using NanoDrop, and serial dilution of mRNA to 250 and 50 ng/μL was performed with RNAase‐free water. Then, the purified mRNA was denatured at 95°C for 15 min, followed by chilling on ice. Two microlitres of denatured RNA samples was applied to Hybond‐N+ membrane (GE, RPN303B). After UV cross‐linking on Stratalinker 2400, the membrane was washed in 1× Phosphate Buffered Saline with Tween 20 buffer, blocked in 5% skim milk and later incubated with anti‐m^6^A antibody overnight at 4°C. Afterwards, the membrane was washed with 1× TPST buffer and sequentially incubated with goat anti‐rabbit IgG‐Horseradish peroxidase (IgG‐HRP) antibody and Enhanced Chemiluminescence (ECL) solution for chemiluminescence reading on a Chemi system (Bio‐Rad). Meanwhile, membrane was stained with .02% methylene blue, serving as a reference. For colorimetric quantification of m^6^A RNA methylation, the RNA m^6^A level was detected by using the EpiQuikTM m^6^A RNA Methylation Kit (Epigentek, P‐9005‐96) following the manufacturer's protocol.

### 2.12 Analysis of intracellular metabolites

For the determination of ATP levels, the amount of ATP was measured with an ATP Detection Assay Kit (Cayman, 700410) according to the assay protocol. For the assay of cellular glycolysis, glycolytic activity was measured by using Glycolysis Cell‐Based Assay Kit (Cayman, 600450), which allows for colorimetric detection of L‐lactate, the end product of glycolysis, produced and secreted by cultured cells. For the measurement of the cellular glucose uptake, Glucose Uptake Cell‐Based Assay Kit (Cayman, 600470) was employed for measuring the cellular glucose uptake. This kit employs 2‐Deoxy‐2‐[(7‐nitro‐2,1,3‐benzoxadiazol‐4‐yl)amino]‐D‐glucose (2‐NBDG), a fluorescently labelled deoxyglucose analogue, as a probe for the detection of glucose taken up by cultured cells. The level of cellular glutamine was determined with Glutamine Colorimetric Assay Kit (BioVision, K556‐100). Cellular α‐ketoglutarate assay was detected by using Ketoglutarate Colorimetric/Fluorometric Assay Kit (BioVision, K677‐100) according to the assay protocol. To measure the activity of lactate dehydrogenase (LDH), LDH Activity Colorimetric Assay Kit (BioVision, K726‐500) was utilised to quantify LDH activity in cells.

### 2.13 Metabonomic analysis

The extraction solution (acetonitrile:methanol:water = 2:2:1) containing an isotopide‐labelled internal standard mixture was added to the sample, which was then vortexed for 30 s, frozen–thawed three times in liquid nitrogen and sonicated on ice for 10 min. The samples were incubated at –40°C for 1 h, then centrifuged at 12 000 rpm for 15 min at 4°C and 200 μL of the supernatant was blown with nitrogen. The resulting supernatant was transferred to a fresh glass vial for liquid chromatography–mass spectrometry (LC–MS) analysis. LC–MS/MS analyses were performed on a UHPLC system (Vanquish, Thermo Fisher Scientific) with a UPLC BEH Amide column coupled to a Q Exactive HFX mass spectrometer (Orbitrap MS, Thermo). The raw data were converted to the mzXML format using ProteoWizard and processed with an in‐house program, which was developed using R and was based on XCMS.

### 2.14 RNA‐sequencing

Total RNA was extracted from tumour cells (2 × 10^6^) using RNAiso Reagent (Takara), and strand RNA‐sequencing (RNA‐seq) libraries were prepared using the KCTM Stranded mRNA Library Prep Kit for Illumina Hiseq X 10 sequencer platform based on Seqhealth Co., Ltd. according to the protocol recommended by the vendor. FeatureCounts (Subread‐1.5.1; Bioconductor) calculated readings were mapped to the exonic regions of each gene and then Reads Per Kilobase Millions (RPKMs) were calculated. The edgeR package (version 3.12.1) was used to identify differentially‐expressed genes between the groups.

### 2.15 MeRIP‐sequencing

Total RNA was extracted using RNAiso Reagent (Takara). NanoDrop 2000 (Thermo Fisher) was used for qualitative and quantitative analyses of the total RNA. Intact mRNA was isolated from total RNA using the Dynabeads mRNA Purification Kit and then fragmented into ∼100‐nt‐long oligonucleotides using a fragmentation buffer. Fragmented mRNA was co‐immunoprecipitated by incubation with m^6^A‐specific antibody (Synaptic Systems, 202003) overnight at 4°C. Then, the Splint connector was connected, the connector products were purified by magnetic beads and PCR amplified, the quality of the constructed library was checked by agarose electrophoresis, and the library was quantified by Qubit 3.0 to determine whether the concentration of the library was suitable. Immunoprecipitated RNA fragments and comparable amounts of input were subjected to sequencing library construction using the NEBNext Ultra RNA Library Prep Kit (New England Biolabs, E7530). Sequencing was performed on an Illumina HiSeq 2500 platform.

### 2.16 TNBC organoid culture

TNBC tissues were cut into small pieces and mechanically homogenised. The homogenised tissue was digested with trypsin on ice. After centrifugation, single‐cell suspensions were added dropwise with cold 3D Matrigel matrix (Corning, 354348) to six‐well culture plates, which were kept inverted in a cell incubator for 30 min before organoid‐specific medium was added. Organoids were cultured with media containing 1% GlutaMax (Thermo Fish, 35050061), 100 μg/mL Primocin (InvivoGen, 26‐69‐PM), .5 μg/mL hydrocortisone (MedChemExpress, HY‐N0583), 10 μM Y27632 (Sigma, Y0503), .2 nM Wnt3a (StemRD, W3a‐H‐025), 250 ng/mL R‐spondin1 (PeproTech, 120‐38), 100 ng/mL Noggin (PeproTech, 120‐10D), 1× B27 + Vitamin A (Invitrogen, 17504‐044), 10 mM HEPES (Thermo Fish, 15630080), 20 ng/mL Fibroblast Growth Factor‐10 (FGF‐10) (Peprotech, 100‐26), 5 ng/mL Epidermal Growth Factor (EGF) (Peprotech, GMP100‐15), 10 mM nicotinamide (Selleckchem, S1899), 100 nM β‐estradiol (Sigma, E2758), 10 μM Forskolin (MedChemExpress, HY‐15371), 5 nM Heregulin B1 (ProSpec, CYT‐733), .5 μM A83‐01 (MedChemExpress, HY‐10432), .5 μM SB202190 (Selleckchem, S10077), 1.25 mM N‐acetylcysteine (Sigma, A0737) and Dulbecco's Modified Eagle Medium (DMEM).

### 2.17 TNBC patient‐derived xenograft

NOD.Cg‐PrkdcscidIL2rgtm1Wjl/SzJ (NSG) mice were used to establish a TNBC patient‐derived xenograft (PDX) model. Animal experiments were approved by the Institutional Animal Care and Use Committee, Huazhong University of Science and Technology, and conducted in strict accordance with the Institutional Guidelines and Protocols. Briefly, fresh tumour tissues were collected from two TNBC patients after surgical resection at the Wuhan Union Hospital and stored in iced DMEM. Primary TNBC specimens were cut into 2–3 mm^3^ pieces, and mixed with an equal volume of Matrigel matrix (Corning, 354348), and then subcutaneously implanted into the flanks of NSG mice. When the tumours grew to approximately 50–100 mm^3^ in size after injection of cancer cells, mice were randomly divided into four groups (*n* = 5 per group): an FX‐11 treatment group that received intraperitoneal injection of 2 mg/kg FX‐11 daily, a 2‐(4‐Benzylpiperazin‐1‐yl)‐4,5‐diphenyl‐1H‐imidazole (BPTES) treatment group that was intraperitoneally injected with 12.5 mg/kg BPTES daily, an FX‐1 plus BPTES group that was administered daily intraperitoneal injection of FX‐11 (2 mg/kg) and BPTES (12.5 mg/kg), and a control group that was intraperitoneally given normal saline for 3 weeks. The tumour weight and volume were recorded. Tumour volumes were calculated using the following equation: tumour volume (mm^3^) = (length × width^2^)/2. Tumours were harvested for further analysis.

### 2.18 RNA immunoprecipitation assay

All the specific manipulations were performed according to the protocol of Magna RIP RNA‐Binding Protein Immunoprecipitation Kit (Merck Millipore, 17‐700). Briefly, cells (2.0 × 10^7^) were lysed with lysis buffer. Then, 5 μg LRPPRC (Proteintech, 21175‐1‐AP) antibody and control rabbit IgG was conjugated to protein A/G magnetic beads for 4 h at 4°C, followed by incubation with lysate in RNA immunoprecipitation (RIP) buffer at 4°C overnight. The beads were then resuspended in Phosphate Buffered Saline (PBS), and incubated with 50 μg proteinase K for 15 min at 37°C. RNAs were extracted with RNAiso Reagent and subjected to qPCR analysis. The primers for RT‐qPCR are listed in Table S2.

### 2.19 MeRIP–RT‐qPCR

All the specific manipulations for MeRIP assays were performed according to the protocol of Magna MeRIP m6A Kit (Merck Millipore, 17‐10499). Briefly, total RNA was extracted from tumour cells (2 × 10^7^) using RNAiso Reagent (Takara), and fragmented for 5 min at 94°C. Fragmented RNA concentration was measured on a NanoDrop spectrophotometer and the size distribution was checked by agarose gel. Immunoprecipitated magnetic beads were prepared by incubating 50 μL Magna ChIP Protein A/G Magnetic Beads with 10 μg anti‐m^6^A antibody for 30 min at room temperature with rotation. Then, 500 μL MeRIP reaction mixture was added to each bead antibody, and incubated with rotation for 2 h at 4°C. The bound RNA was eluted with 100 μL of elution buffer. The eluted RNA was purified with a RNeasy MiniElute Cleanup kit (QIAGEN, 74204) and then subjected to qPCR with One Step TB Green PrimeScript RT‐PCR Kit (Takara, RR066A). The primers for RT‐qPCR are listed in Table S2.

### 2.20 RNA pull‐down assay

Biotin‐labelled RNAs were synthesised by Tsingke. RNA probes included probes that contained an m^6^A base or an adenine base mutation or a guanine base mutation. The RNA pull‐down assay was performed by utilising Pierce Magnetic RNA–Protein Pull‐Down Kit (Thermo Fisher, 20164). Briefly, cells were lysed in 400 μL of IP lysis buffer containing protease/phosphatase inhibitor cocktail and RNase inhibitor. One‐tenth of the lysate was retained as input. To the remaining supernatant, 50 μL of biotinylated RNAs and streptavidin agarose beads mixture was added and the resultant sample was incubated for 2 h at room temperature. After five thorough washes, the RNA–protein binding mixture was eluted. The eluted protein samples were denatured with SDS buffer and detected by Western blotting.

### 2.21 mRNA stability assay

Cells were treated with actinomycin D (Sigma–Aldrich, A9415) at a concentration of 5 mg/mL to derepress global mRNA transcription. Then, cells were lysed at the indicated times and total RNA was extracted for reverse transcription. The cellular mRNA transcript levels were measured by qPCR.

### 2.22 Extracellular acidification rate

Extracellular acidification rate (ECAR) was measured by using the Glycolysis Stress Test kit (Agilent Technologies, 103020‐100) according to the assay protocol on a Seahorse XFe24 Extracellular Flux Analyser (Agilent Technologies). Briefly, after washing the cells, they were resuspended in the culture medium supplemented with 2 mM glutamine, and incubated for 30 min without CO_2_. Glycolysis was measured by adding glucose (10 mM), oligomycin (1.5 μM) and 2‐deoxy‐D‐glucose (50 mM).

### 2.23 In vivo assays of tumourigenesis and metastasis assays

For tumourigenesis assay, BT549 cells (5 × 10^6^) stably overexpressing LRPPRC, MDA‐MB‐231 cells (5 × 10^6^) with LRPPRC stably knocked down and control cells were suspended in 100 μL PBS and injected subcutaneously into 6–8 weeks old BALB/c nude mice (*n* = 4 per group), respectively. When the tumour grew to 50–100 mm^3^, the tumour‐bearing mice with stable LRPPRC overexpression and the controls were intraperitoneally injected FX‐11 (2 mg/kg body weight), and the tumour‐bearing mice with LRPPRC stably knocked down and the controls were intraperitoneally given BPTES (12.5 mg/kg body weight). The mice were monitored by daily palpation for tumour formation, and tumour size was calculated by using the following equation: *V* = (length × width^2^)/2. After 7–8 weeks, mice were sacrificed and xenografts were weighed.

For metastasis assay, BT549 cells stably overexpressing LRPPRC (5 × 10^5^), MDA‐MB‐231 cells with LRPPRC stably knocked down (5 × 10^5^) and control cells were suspended in 100 μL PBS and injected into the tail vein of 6–8 weeks old BALB/c nude mice (*n* = 4), respectively. Mice were treated with the aforementioned drugs. After 6 weeks, mice were sacrificed and the number of metastatic nodules was counted.

### 2.24 Study approvals

The experimental mice were housed in a specific pathogen‐free facility of the Huazhong University of Science and Technology, and all procedures were performed in strict accordance with the guidelines of the Institutional Animal Care and Use Committee, Huazhong University of Science and Technology (S2815), Wuhan, China. Human tumour tissues were obtained from Wuhan Union Hospital, Huazhong University of Science and Technology, Wuhan, China. The study was approved by the Institutional Human Ethics Committee of Tongji Medical College, Huazhong University of Science and Technology (S093), Wuhan, China. Informed consent was obtained from all tissue donors.

### 2.25 Statistical analysis

GraphPad Prism software 8.0 was used for statistical analysis of data. The difference of measurement data was compared by using the Student's *t*‐test and analysis of variance, respectively. Statistical significance was set at a *p* < .05 and was denoted as follows: ^****^ *p* < .0001; ^***^ *p *< .001; ^**^ *p* < .01; ^*^ *p* < .05.

## 3 RESULTS

### 3.1 LRPPRC is upregulated and binds to m^6^A in TNBC

**FIGURE 1:**
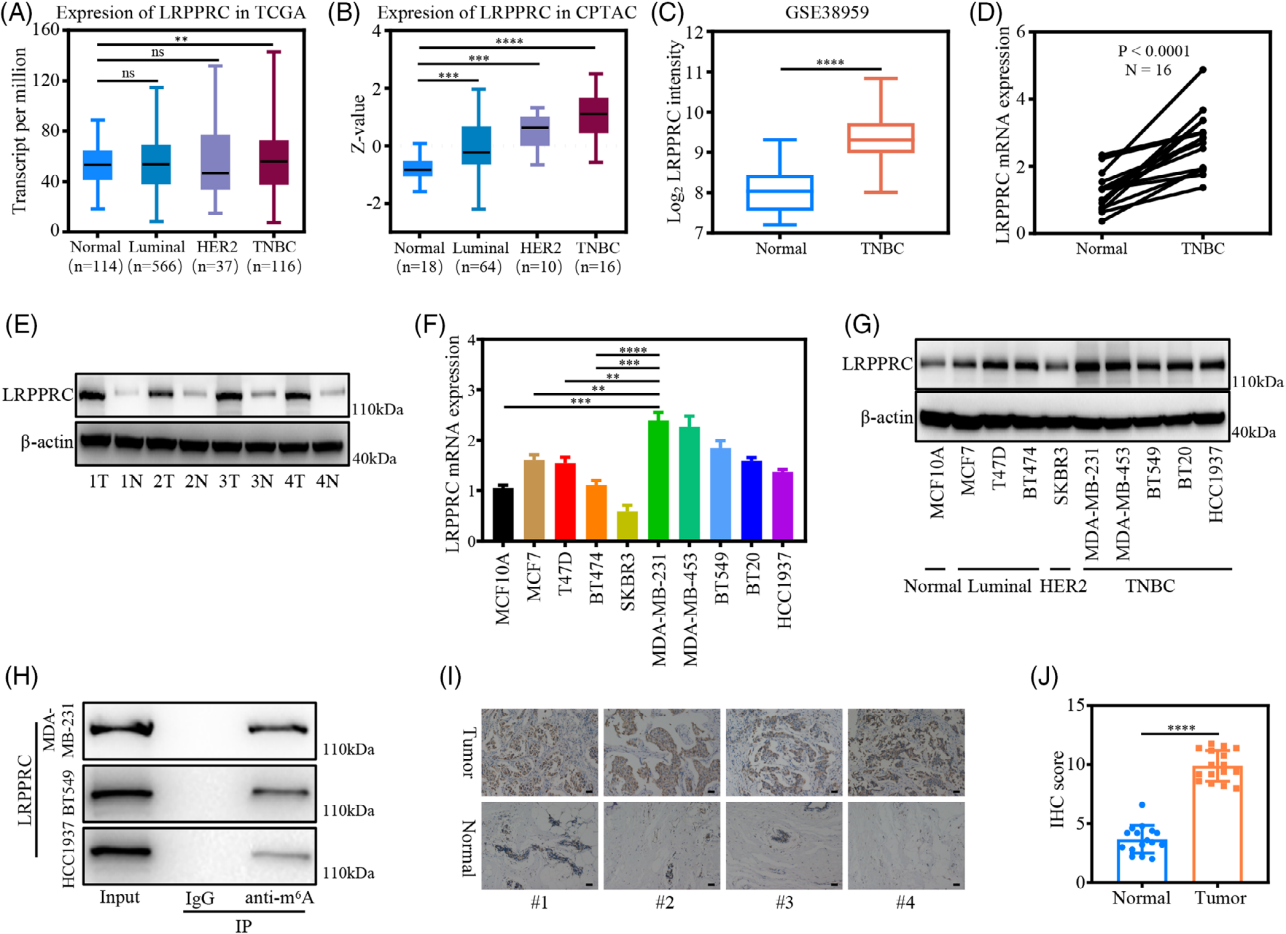
Expression of leucine‐rich pentatricopeptide repeat containing protein (LRPPRC) in triple‐negative breast cancer (TNBC) and its ability to bind to N6‐methyladenosine (m^6^A) modification sites. (A and B) Relative mRNA and protein expression levels of LRPPRC in breast cancer subtypes based on TCGA and CPTAC datasets, respectively. (C) Relative mRNA levels of LRPPRC in TNBC and adjacent normal tissues in the GEO dataset. (D) qRT‐PCR analysis of LRPPRC mRNA expression in TNBC tissues and adjacent normal tissues (*n* = 16). (E) Expression of LRPPRC in 16 pairs of TNBC tissues and adjacent normal tissues were detected by Western blotting. (F and G) qRT‐PCR and Western blotting of the expression levels of LRPPRC in normal breast cell line and breast cancer cell lines, respectively. (H) Co‐immunoprecipitation (Co‐IP) and Western blot assays revealing the interaction between endogenous LRPPRC with m^6^A modification sites in TNBC cells. Western blot images are representative of three independent experiments. (I) Representative immunohistochemical (IHC) images of LRPPRC in four pairs of TNBC tissues and adjacent normal tissues. Scale bar, 20 μm. (J) IHC scores of LRPPRC in human TNBC cohort (*n* = 16). Western blot images are representative of three independent experiments. Values are the mean ± standard deviation (S.D.) of *n* = 3 independent experiments.

Recent studies have shown that LRPPRC could act as an m^6^A ‘reader’.^16^ Therefore, we examined the expression of LRPPRC mRNA in breast cancer by searching The Cancer Genome Atlas (TCGA) database. Notably, LRPPRC mRNA expression was elevated in breast cancer tissues relative to that in normal tissues (Figure S1A). Interestingly, the expression level of LRPPRC mRNA was specifically upregulated in TNBC compared to other breast cancer subtypes (Figure 1A). We then examined the expression of the LRPPRC protein in breast cancer by searching Clinical Proteomic Tumor Analysis Consortium (CPTAC) database. Compared to normal tissues, the expression level of LRPPRC protein was also increased in breast cancer tissues (Figure S1B). Moreover, LRPPRC protein expression was significantly higher in TNBC than in other breast cancer subtypes (Figure 1B). Meanwhile, we retrieved the expression pattern of LRPPRC in TNBC from Gene Expression Omnibus (GEO) datasets (GSE38599) and found that LRPPRC was upregulated in TNBC compared to normal breast cells (Figure 1C). Next, we verified these findings in 16 pairs of in‐house TNBC tissues and matched them with normal tissues using qRT‐PCR and Western blotting (Figures 1D,E and S1C). LRPPRC mRNA and protein levels were significantly elevated in TNBC. Simultaneously, we detected the expression of LRPPRC in normal breast epithelial cell line and breast cancer cell lines. Consistent with the aforementioned results, qRT‐PCR and Western blotting showed that LRPPRC was highly expressed in TNBC cell lines relative to normal breast cell lines and cell lines of other breast cancer subtypes (Figure 1F,G).

To understand the roles of m^6^A modification in TNBC, we detected m^6^A RNA levels in 18 TNBC and matched normal tissues. Dot‐blot assay exhibited that m^6^A RNA levels were significantly higher in most TNBC tissues (Figure S1D). We also determined m^6^A RNA levels in normal breast epithelial cell lines and breast cancer cell lines and found that m^6^A RNA levels were elevated in most TNBC cell lines (Figure S1E). The results were also confirmed by colorimetric ELISA using the m^6^A RNA methylation quantification kit (Figure S1F). We further verified that LRPPRC could bind to m^6^A modification sites in TNBC. Co‐IP assays demonstrated that m^6^A modification sites could interact with LRPPRC in MDA‐MB‐231, BT549 and HCC1937 cells (Figure 1H). Confocal microscopy further revealed that LRPPRC and m^6^A modification sites colocalised in the cytoplasm of MDA‐MB‐231 and BT549 cells (Figure S1G). IHC analysis demonstrated that LRPPRC protein expression was upregulated and cytoplasmically sublocalised in TNBC cells (Figures 1I,J and S1H).

### 3.2 LRPPRC promotes TNBC progression and metastasis in vitro

**FIGURE 2:**
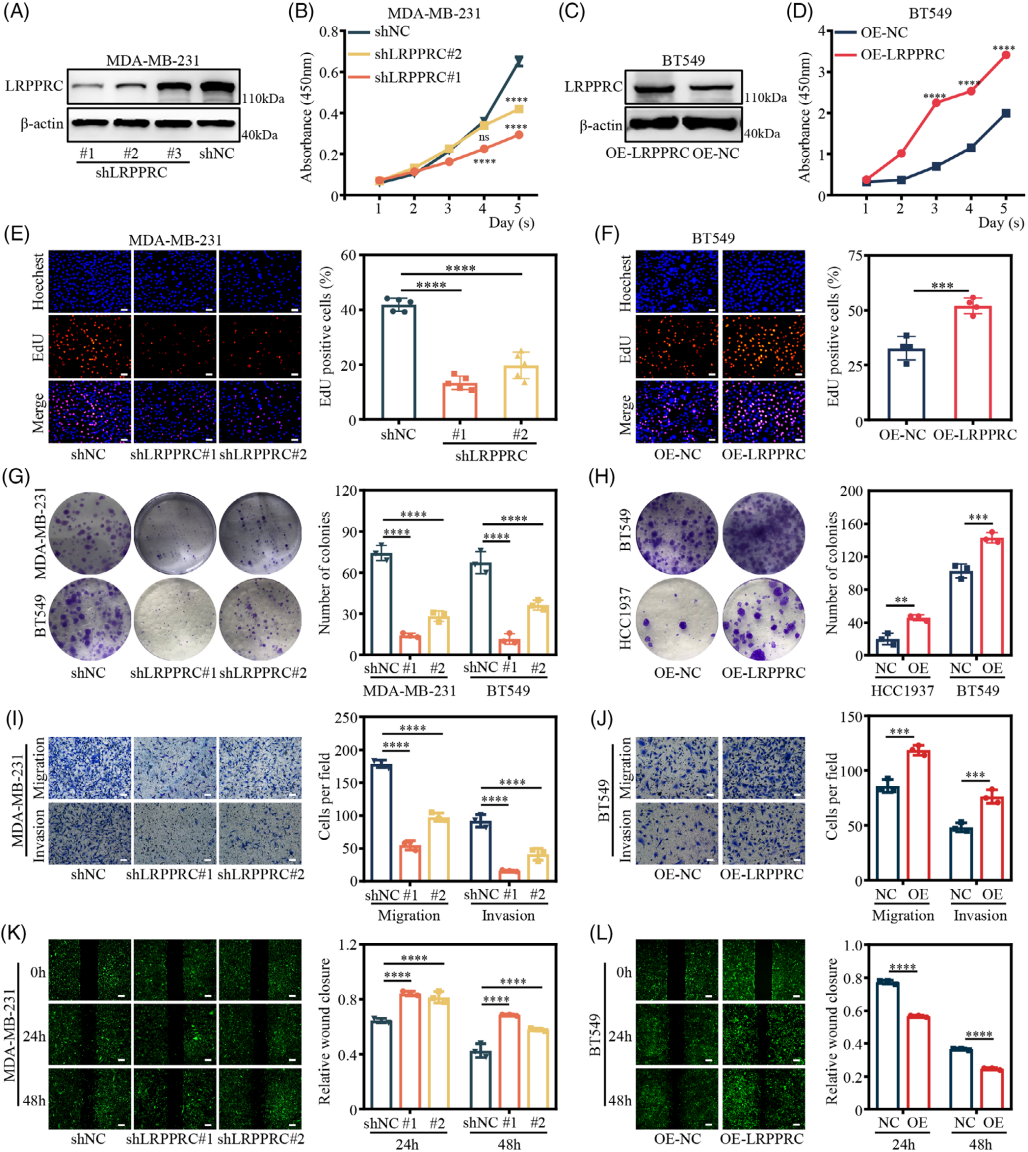
Leucine‐rich pentatricopeptide repeat containing protein (LRPPRC) promoted the proliferation, migration and invasion of triple‐negative breast cancer (TNBC) cells in vitro. (A) Decreased LRPPRC expression in MDA‐MB‐231 transfected with LRPPRC‐inhibiting shRNAs as exhibited by Western blot analysis. (B) Cell growth after LRPPRC knockdown in MDA‐MB‐231 cells as shown by cell counting kit‐8 (CCK‐8) assays. (C) Elevated LRPPRC expression in BT549 transfected with plasmids overexpressing LRPPRC as exhibited by Western blotting. (D) Cell growth after LRPPRC overexpression in BT549 cells as determined by CCK‐8 assays. (E and F) Cell growth after LRPPRC knockdown MDA‐MB‐231 and LRPPRC overexpression BT549 cells described in (A and C), respectively, as shown by EdU assays. Representative images are shown in the left panel. Scale bar, 50 μm. Quantification of EdU‐positive cells are shown in the right panel. (G and H) Cell growth after LRPPRC knockdown MDA‐MB‐231 and LRPPRC overexpression of BT549 cells described in (A and C), respectively, as determined by colony formation assays. (I and J) Effects of LRPPRC on migration and invasive abilities of LRPPRC‐knocked down MDA‐MB‐231 and LRPPRC‐overexpressing BT549 cells described in (A and C), respectively, as determined by transwell invasion assays. Scale bar, 50 μm. (K and L) Effects of LRPPRC on migration abilities of LRPPRC‐knocked down MDA‐MB‐231 and LRPPRC‐overexpressing BT549 cells described in (A and C), respectively, as determined by wound healing assays. Scale bar, 50 μm. Values are the mean ± standard deviation (S.D.) of *n* = 3 independent experiments.

To explore the function of LRPPRC in TNBC in vitro, we characterised the phenotypes of TNBC cells upon depletion or overexpression of LRPPRC. The effect of LRPPRC knockdown was confirmed by establishing two stable shRNA‐expressing (shLRPPRC#1 and shLRPPRC#2) human TNBC cell lines, that is, MDA‐MB‐231 and BT549 (Figures 2A and S2A,B,E). The effect of LRPPRC knockdown was also substantiated by generating two stable LRPPRC‐overexpressing (OE‐LRPPRC) human TNBC cell lines, BT549 and HCC1937 (Figures 2C and S2C,D,G). CCK‐8 (Figures 2B and S2F), EdU staining (Figures 2E and S2I) and colony formation (Figure 2G) assays showed that LRPPRC deficiency significantly repressed the proliferation and colony formation of cells compared to the control groups. Additionally, we investigated the role of LRPPRC in the metastatic capacity of TNBC cells. Both transwell assays (Figures 2I and S2K) and wound healing (Figures 2K and S2M) indicated that the cells with LRPPRC knocked down exhibited less aggressive migratory and invasive potential. Reciprocally, LRPPRC overexpression significantly promoted the growth of BT549 and HCC1937 cells, as shown by CCK‐8 (Figures 2D and S2H), EdU staining (Figures 2F and S2J) and colony‐formation assay (Figure 2H). Moreover, the migration and invasion abilities of BT549 and HCC1937 cells were markedly enhanced following LRPPRC overexpression (Figures 2J,L and S2L,N). These results indicated that LRPPRC played an important role in the proliferation, migration and invasion of TNBC cells.

### 3.3 LRPPRC promotes glycolysis in TNBC

**FIGURE 3:**
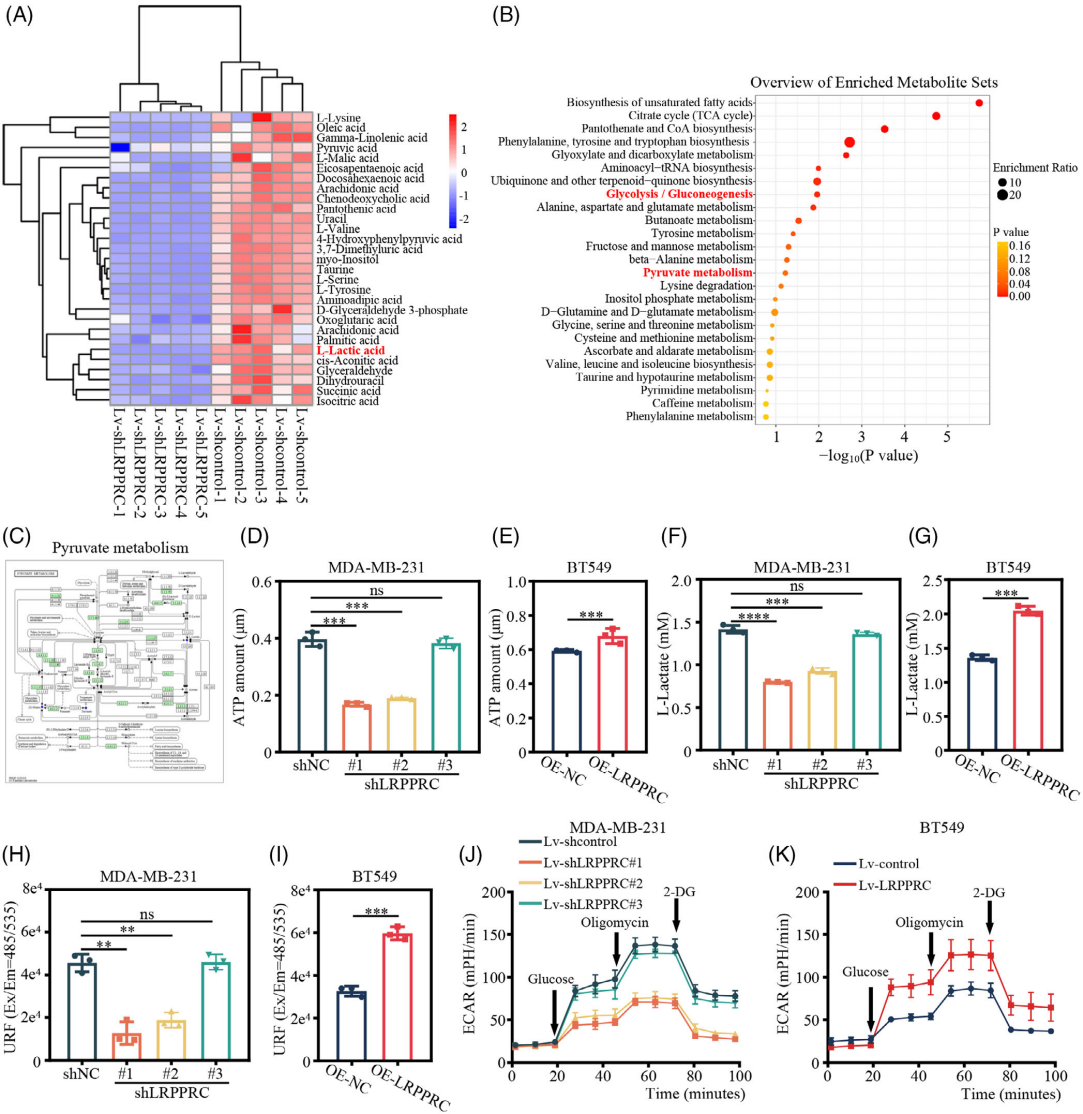
Leucine‐rich pentatricopeptide repeat containing protein (LRPPRC) promoted glycolysis in triple‐negative breast cancer (TNBC). (A) The hierarchical clustering heatmap of differential metabolites of the control and LRPPRC‐knocked down MDA‐MB‐231 cells in positive ion mode. (B) Metabolic pathway enrichment analysis of differential metabolites described in (A). (C) KEGG enrichment analysis was used to analyse the differential metabolites between control and knockdown LRPPRC MDA‐MB‐231 cells. Blue dots represent the differentially‐expressed compounds. (D and E) Intracellular ATP levels in LRPPRC‐knocked down MDA‐MB‐231 (D) and LRPPRC‐overexpressing BT549 (E) cells, respectively. (F and G) Glycolysis cell‐based assays of LRPPRC‐knocked down MDA‐MB‐231 (F) and LRPPRC‐overexpressing BT549 (G) cells, respectively. L‐lactate was detected as the end product of glycolysis. (H and I) Glucose uptake cell‐based assays of LRPPRC‐knocked down MDA‐MB‐231 (H) and LRPPRC‐overexpressing BT549 (I) cells, respectively. (J and K) Extracellular acidification rate (ECAR) measured in LRPPRC‐knocked down MDA‐MB‐231 (J) and LRPPRC‐overexpressing BT549 (K) cells, respectively. Basal ECAR measurement was measured in extracellular flux (XF) assay medium without glucose, following by the addition of glucose (10 mM), oligomycin (1.5 μM) and 2‐deoxy‐D‐glucose (2‐DG) (50 mM). Values are the mean ± standard deviation (S.D.) of *n* = 3 independent experiments.

Previous studies have shown that LRPPRC plays an important part in the regulation of energy metabolism.^15^ A question remains whether LRPPRC is implicated in the regulation of energy metabolism in TNBC. During the culture of TNBC cells with LRPPRC stably knocked down or LRPPRC stably overexpression, we found that phenol red in the cell culture medium exhibited a gradual change from red to yellow at lower pH values and this might be due to lactate accumulation^17^ (Figure S3A). Moreover, the acidity of the culture medium decreased after LRPPRC knockdown, while the opposite results were observed in LRPPRC‐overexpressing cells (Figure S3B,C). Then, we wondered whether LRPPRC plays a role in the regulation of glucose metabolism in TNBC. LC–MS/MS was performed to examine the differences in metabolite changes between MDA‐MB‐231 cells with LRPPRC knockdown and control cells. The hierarchical clustering analysis demonstrated that in LRPPRC‐knocked down cells, some of the differential metabolites (Figures 3A and S3D–G), had significantly reduced expression of L‐lactic acid, and metabolic pathway enrichment analysis showed that the differential metabolites were enriched in ‘glycolysis or gluconeogenesis’ and ‘pyruvate metabolism’ pathways both in positive and negative ion modes (Figure 3B). Kyoto Encyclopedia of Genes and Genomes (KEGG) pathway annotation showed that these differential metabolites also clustered into the ‘pyruvate metabolism’ pathway (Figures 3C and S3H). In view of these findings, our focus was directed at looking into the effect of LRPRPC on glycolysis in TNBC.

To confirm the biological effect of LRPPRC on glycolysis in TNBC, we investigated the glycolysis by determining the ATP content, lactate production and glucose uptake. LRPPRC knockdown resulted in a significant decrease in ATP levels, lactate production and glucose consumption, whereas overexpression of LRPPRC led to an opposite effect in TNBC cells (Figures 3D–I and S3I–N). We then measured ECAR in TNBC cells, which is indicative of glycolysis level. The results showed that LRPPRC knockdown lowered glycolysis rate and capacity, whereas overexpression of LRPPRC led to an increase in ECAR (Figure 3J,K).

### 3.4 LRPPRC promotes LDHA mRNA stability and expression in an m^6^A‐dependent manner

To fully understand the mechanisms underlying the oncogenic role of LRPPRC in TNBC progression, we performed RNA‐seq analysis on LRPPRC knockdown and control MDA‐MB‐231 cells. LRPPRC deficiency resulted in global alterations in 1716 genes, including 1107 upregulated genes and 609 downregulated genes (Figure S4A). Gene ontology analysis indicated that those downregulated genes were enriched for pyruvate and lactate metabolic processes (Figure S4B). Meanwhile, in the KEGG pathway annotation, downregulated genes were also enriched in the ‘pyruvate metabolism’ and ‘glycolysis’ pathways (Figure S4C). The consistency of the RNA analysis findings with metabolomic results suggests that LRPPRC plays an important role in the glycolysis in TNBC.

**FIGURE 4:**
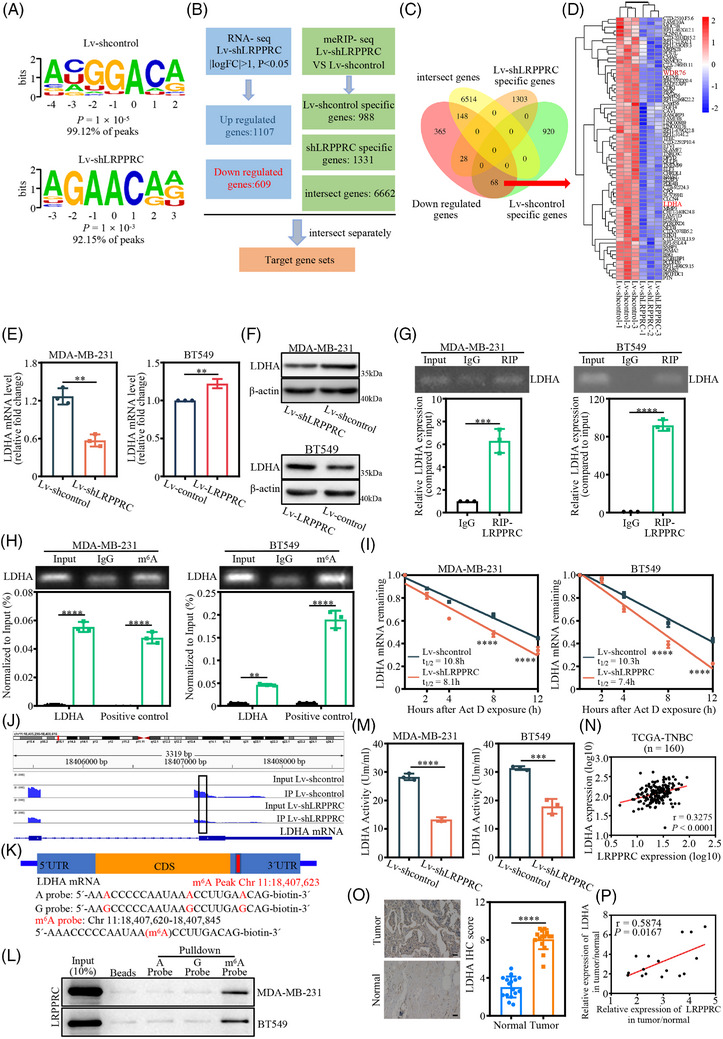
Lactate dehydrogenase A (LDHA) is a key target of leucine‐rich pentatricopeptide repeat containing protein (LRPPRC) in triple‐negative breast cancer (TNBC). (A) Enriched top motifs identified by HOMER with N6‐methyladenosine‐sequencing (m^6^A‐seq) peaks in LRPPRC‐knocked down and control MDA‐MB‐231 cells. (B) Schematic representation of downstream target analysis for MeRIP‐sequencing (MeRIP‐seq) and mRNA‐sequencing (mRNA‐seq). (C) Venn diagram shows the three groups of genes enriched by MeRIP‐seq and the downregulated genes enriched by RNA‐seq in LRPPRC‐knocked down and control MDA‐MB‐231 cells. (D) Heatmap shows downregulated genes described in Venn diagram. (E) The mRNA expression of LDHA upon LRPPRC knockdown in MDA‐MB‐231 and LRPPRC overexpression in BT549 cells, respectively, as revealed by qRT‐PCR. (F) LDHA expression upon LRPPRC knockdown in MDA‐MB‐231 and LRPPRC overexpression in BT549 cells, respectively, as displayed by Western blotting. Western blot images are representative of three independent experiments. (G) RNA immunoprecipitation (RIP)–PCR confirming LRPPRC binding to LDHA mRNA in MDA‐MB‐231 and BT549 cells. (H) Gene‐specific m^6^A qPCR validation of m^6^A levels on LDHA mRNA in MDA‐MB‐231 and BT549 cells. (I) The levels of LDHA expression in LRPPRC‐knocked out and control TNBC cells treated with actinomycin D (5 μg/mL) at the indicated time points were detected by qRT‐PCR. (J) m^6^A abundance in LDHA mRNA transcripts in LRPPRC‐knocked down (immunoprecipitation [IP] and input) cells and negative control (IP and input). The mapped reads represent enriched RNA fragments by MeRIP experiment loaded by Integrative Genomics Viewer (IGV). (K) Position of m^6^A peak in LDHA mRNA (upper panel); RNA probe sequences for RNA pull‐down (lower panel). (L) LRPPRC recognises the m^6^A site in the 3′UTR of LDHA mRNA as shown by RNA pull‐down assays. (M) Colorimetrically‐determined lactate dehydrogenase activity in of LRPPRC‐knocked down MDA‐MB‐231 and BT549 cells, respectively. (N) The correlation between LRPPRC and LDHA in the TCGA TNBC database (Pearson's correlation). (O) Representative immunohistochemical (IHC) images of LDHA in pairs of matched TNBC tissues and adjacent normal tissues. Scale bar, 20 μm (left panel); IHC scores of LRPPRC in human TNBC cohort (*n* = 16) (right panel). (P) Correlation between relative LRPPRC and LDHA protein abundances in 16 pairs of matched TNBC tumour and adjacent normal tissues (Pearson's correlation). Values are the mean ± standard deviation (S.D.) of *n* = 3 independent experiments.

Recent studies have shown that LRPPRC acts as an m^6^A reader and functions by binding and affecting m^6^A‐methylated transcripts. We then identified potential transcripts with m^6^A modification that were regulated by LRPPRC using MeRIP‐seq in LRPPRC‐knocked down and control MDA‐MB‐231 cells. MeRIP‐seq identified m^6^A peaks in 7993 and 7561 genes in LRPPRC‐knocked down cells and control cells, respectively. GRACA was the most enriched motif in m^6^A peaks in both LRPPRC‐knocked down cells and control MDA‐MB‐231 cells (Figure 4A). The m^6^A peaks were predominantly situated in protein‐coding transcripts and enriched near the stop codons in LRPPRC‐knocked down and control cells (Figure S4D,E). Upon LRPPRC knockdown, possibly due to changes in gene expression levels, 988 genes showed reduced m^6^A modification (that is, shNC‐specific genes), and 1331 genes showed increased m^6^A modifications (i.e., shLRPPRC‐specific genes). Based on the above cell phenotypic changes in glucose metabolism and RNA‐seq data, we identified 68 candidate genes with reduced mRNA levels and m^6^A modification upon LRPPRC knockdown (Figure 4B,C). Importantly, we found that LDHA, an important player in glycolysis, was the target of LRPPRC (Figure 4D).

We further examined the direct interaction between LRPPRC and its target LDHA transcript. First, qRT‐PCR and Western blotting showed that the expression of LDHA mRNA and protein was significantly downregulated following LRPPRC knockout, and was upregulated after LRPPRC overexpression (Figures 4E,F and S4F,G). Additionally, RIP–RT‐qPCR also showed that, in TNBC cells, the LRPPRC antibody conspicuously enriched the mRNA of LDHA compared to the IgG pull‐down group (Figure 4G), confirming a direct interaction between LRPPRC and LDHA. MeRIP–RT‐qPCR further revealed abundant m^6^A modifications in LDHA mRNA and a significant enrichment of LDHA mRNA in TNBC cells (Figure 4H). Since the mRNA level of LDHA decreased upon LRPPRC knockdown and LRPPRC was localised in the cytoplasm, we speculated that LRPPRC might regulate the stability of LDHA mRNA. RNA decay assay revealed that the stability of LDHA mRNA decreased significantly upon LRPPRC knockdown (Figure 4I). To identify co‐factors of LRPPRC that may enhance stability of mRNA targets, Co‐IP assay demonstrated that LRPPRC could interact with ELAV‐like RNA‐binding protein 1 (ELAVL1; also known as HuR) and poly(A)‐binding protein cytoplasmic 1 (PABPC1), two known mRNA stabilisers in MDA‐MB‐231and BT549 cells (Figure S4H). The spatial distribution of the LDHA mRNA relative to HuR and PABPC1 was also examined by fluorescence in situ hybridisation (FISH) and fluorescence immunostaining in MDA‐MB‐231 and BT549 cells (Figure S4I–K). Next, we examined whether the regulation of LDHA expression by LRPPRC was m^6^A dependent. An obviously lowered m^6^A peak in LDHA mRNA induced by LRPPRC knockdown was visualised with Integrative Genomics Viewer (IGV) (Figure 4J). RNA pull‐down experiments were conducted using an RNA probe for the 3′UTR sequence of LDHA mRNA with an m^6^A site (Figure 4K). We again confirmed that LRPPRC bound to the m^6^A site (Figure 4L). Moreover, the LDHA activity assay showed that LRPPRC knockdown significantly reduced LDHA activity in MDA‐MB‐231 and BT549 cells (Figure 4M). Finally, we determined the LDHA expression and established its association with LRPPRC in TNBC. Analysis of TCGA database showed that LDHA was positively correlated with LRPPRC at the transcriptional level in TNBC (Figure 4N). IHC results showed that LDHA was overexpressed in in‐house TNBC tissues and positively correlated with LRPPRC expression (Figures 4O,P and S5A). Meanwhile, GEO datasets search substantiated the positive correlation among them in TNBC (GSE53572 and GSE62931) (Figure S5B,C). Collectively, these results corroborated that LRPPRC functions as a regulator of LDHA.

### 3.5 LRPPRC promotes TNBC progression through LDHA

To further determine whether LDHA is a direct effector of LRPPPRC in TNBC progression, we conducted a rescue experiment by overexpressing LDHA in LRPPRC‐knocked down MDA‐MB‐231 cells or treating LRPPRC‐overexpressing BT549 cells with an LDHA inhibitor. As shown in Figure S6, ectopic expression of LDHA partially reversed the effects of LRPPRC depletion on proliferation, migration, invasion and ATP levels in MDA‐MB‐231 cells (Figure S6A,C,E,G,I,K). Consistent with these findings, FX‐11, an inhibitor of LDHA, was also found to reverse the phenotype caused by LRPPRC overexpression in BT549 cells (Figure S6B,D,F,H,J,L). Taken together, these data indicated that LDHA, an oncogenic factor, is a functionally critical target of LRPPRC in TNBC.

### 3.6 LRPPRC knockdown decreases WDR76 expression and induces synthetic lethality

**FIGURE 5:**
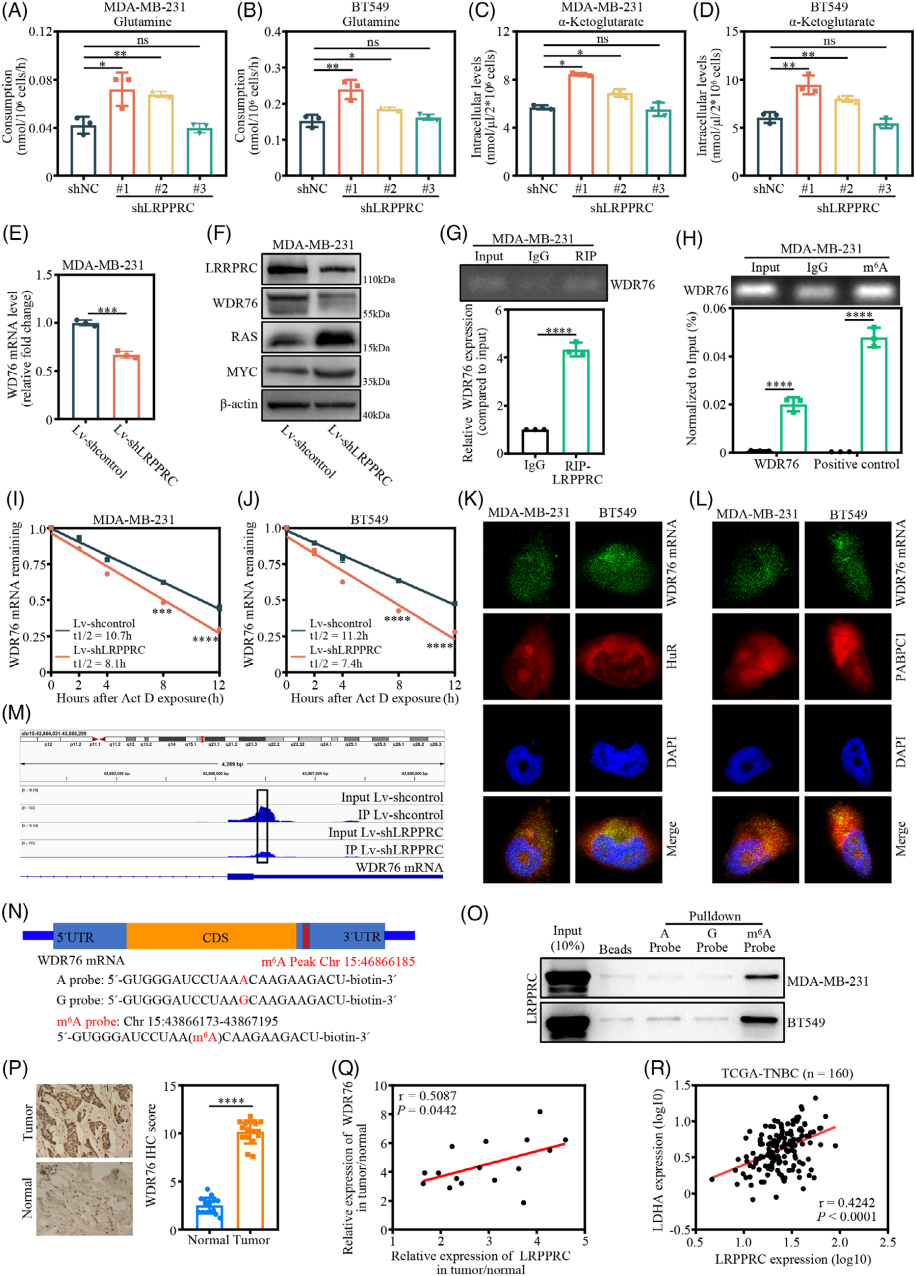
Leucine‐rich pentatricopeptide repeat containing protein (LRPPRC) regulates GPT2 expression via an N6‐methyladenosine (m^6^A)‐dependent manner in triple‐negative breast cancer (TNBC). (A and B) Glutamine cell‐based assays of LRPPRC‐knocked down MDA‐MB‐231 (A) and BT549 (B) cells, respectively. (C and D) α‐Ketoglutarate cell‐based assays of LRPPRC‐knocked down MDA‐MB‐231 (C) and BT549 (D) cells, respectively. (E) The mRNA expression of WD40 repeat domain‐containing protein 76 (WDR76) upon LRPPRC knockdown in MDA‐MB‐231 by qRT‐PCR. (F) WDR76, RAS and MYC expression upon LRPPRC knockdown in MDA‐MB‐231 by Western blotting. (G) RNA immunoprecipitation (RIP)–PCR validating LRPPRC binding to GPT2 mRNA in MDA‐MB‐231. (H) Gene‐specific m^6^A qPCR validation of m^6^A levels on GPT2 mRNA in MDA‐MB‐231. (I and J) The levels of WDR76 expression in LRPPRC‐knocked down MDA‐MB‐231 (M) and BT549 (N) cells treated with actinomycin D (5 μg/mL) at the indicated time points were detected by qRT‐PCR, respectively. (K and L) Fluorescence in situ hybridisation of WDR76 mRNA and fluorescence immunostaining of HuR (K) or PABPC1 (L) in MDA‐MB‐231 and BT549 cells. Images are representative of three independent experiments. (M) m^6^A abundances in WDR76 mRNA transcripts in LRPPRC‐knocked down (immunoprecipitation [IP] and input) and negative control (IP and input). The mapped reads represent enriched RNA fragments by MeRIP experiment loaded by IGV. (N) Position of m^6^A peak in WDR76 mRNA (upper panel); RNA probe sequences for RNA pull‐down (lower panel). (O) LRPPRC recognises the m^6^A site in the 3′UTR of WDR76 mRNA as shown by RNA pull‐down assays. (P) Representative immunohistochemical (IHC) images of WDR76 in pairs of matched TNBC tissues and adjacent normal tissues. Scale bar, 20 μm (left panel); IHC scores of LRPPRC in human TNBC cohort (*n* = 16) (right panel). (Q) Correlation between relative LRPPRC and WDR76 protein abundances in 16 pairs of matched TNBC tumour and adjacent normal tissues. (R) The correlation between LRPPRC and WDR76 in the TCGA TNBC database (Pearson's correlation). Western blot images are representative of three independent experiments. Values are the mean ± standard deviation (S.D.) of *n* = 3 independent experiments.

It has been reported that Warburg effect supports oncogenesis by coupling pyruvate production to glutamine metabolism.^18^ Moreover, LRPPRC knockdown reportedly could significantly suppress the proliferation of TNBC cells, but no significant cellular death was observed. Therefore, we speculated that the effect of LRPPRC knockdown might be offset by glutamine metabolism after the inhibition of cellular glycolysis. We then assessed the changes in cellular glutamine metabolites upon LRPPRC knockdown in MDA‐MB‐231 and BT549 cells. The results indicated that the cellular glutamine consumption (Figure 5A,B) and α‐ketoglutarate (Figure 5C,D) was increased after LRPPRC knockdown. However, culler alanine levels did not change significantly after LRPPRC knockdown (Figure S7A,B). Furthermore, we treated LRPPRC knockdown MDA‐MB‐231 and BT549 cells with BPTES, an inhibitor of glutaminase. The CCK‐8 assay indicated that the proliferation of TNBC cells was remarkably suppressed upon LRPPRC knockdown (Figure S7C,D). Similarly, colony formation assay revealed that the clonogenic capacity of the cells was inhibited, and the IC50 of BPTES was significantly reduced in LRPPRC knockdown TNBC cells (Figure S7E,F). Meanwhile, we cultured LRPPRC‐knocked down MDA‐MB‐231 and BT549 cells in glutamine‐free medium. Flow cytometry showed that LRPPRC knockdown TNBC cells experienced significantly higher apoptosis in glutamine‐free medium (Figure S7G,H). These results suggested that cell‐dependent glutamine metabolism was increased after LRPPRC knockdown, and that LRPPRC knockdown plus a glutamine inhibitor could induce synthetic lethality.

To explore the mechanisms by which LRPPRC knockdown enhanced glutamine metabolism and LRPPRC knockdown plus glutaminase inhibitors induced synthetic lethality in TNBC cells, we first analysed the RNA‐seq results upon LRPPRC knockout and found that the KEGG pathway was involved in ‘glutamate metabolism’ (Figure S7I). Meanwhile, we found that the expression of WDR76, as a tumour suppressor, was significantly reduced upon LRPPRC knockdown among 68 candidate genes screened (Figure 4C). Furthermore, studies have shown that WDR76 could promote the degradation of RAS protein, which depends on the ubiquitination degradation pathway.^19^ More importantly, studies have shown that RAS enhanced MYC protein stability,^20^ while MYC stimulated glutaminolysis via a transcriptional program.^21^ Thus, since WDR76 plays a regulatory role in the RAS/MYC signalling pathway, we identified WDR76 as a target for enhancing glutamine metabolism upon LRPPRC knockdown. qRT‐PCR and Western blotting showed that the expression of WDR76 mRNA and protein in MDA‐MB‐231 and BT549 cells was significantly decreased after LRPPRC knockdown (Figures 5E,F and S8A,B). RIP–RT‐qPCR also demonstrated that the LRPPRC antibody obviously enriched the mRNA of WDR76 as compared to the IgG pull‐down group in MDA‐MB‐231 and BT549 cells (Figures 5G and S8C), thereby confirming the direct interaction between LRPPRC and WDR76. We then determined the m^6^A modification status of WDR76 mRNA using MeRIP–RT‐qPCR and found significant enrichment of WDR76 mRNA in TNBC cells (Figures 5H and S8D). RNA decay assay showed that the decay of WDR76 mRNA was enhanced after LRPPRC knockdown (Figure 5I,J). The spatial distribution of the WDR76 mRNA relative to HuR and PABPC1 was also examined by FISH and fluorescence immunostaining in MDA‐MB‐231and BT549 cells (Figure 5K,L). An obviously lowered m^6^A peak of WDR76 elicited by LRPPRC knockdown was visualised using IGV (Figure 5M). RNA pull‐down experiments were performed using an RNA probe for the WDR76 mRNA 3′UTR sequence with the m^6^A site (Figure 5N). We demonstrated that LRPPRC bound to WDR76 mRNA m^6^A site (Figure 5O). Finally, we determined the WDR76 expression and established its association with LRPPRC in TNBC. IHC results showed that WDR76 was overexpressed in in‐house TNBC tissues and positively correlated with LRPPRC expression (Figures 5P,Q and S8I). Analysis of TCGA database showed that WDR76 was positively correlated with LRPPRC at the transcriptional level in TNBC (Figure 5R). Moreover, Western blotting showed that the expression of RAS protein in MDA‐MB‐231 and BT549 cells was significantly increased after LRPPRC knockdown (Figures 5F and S8B). Studies have shown that WDR76, as an E3 ubiquitin ligase, mediates the polyubiquitination‐dependent degradation of RAS.^19^ Overexpression of Flag‐WDR76 in MDA‐MB‐231 and BT549 cells reduced expression of the RAS protein in a dose‐dependent fashion (Figure S8E). Furthermore, we examined the half‐life of the RAS protein in the presence of WDR76 upon treatment with the de novo protein synthesis inhibitor cycloheximide. The half‐life of endogenous RAS was remarkably decreased in the WDR76‐overexpressing MDA‐MB‐231 cell line (Figure S8F). Next, we investigated whether the ubiquitination activity of WDR76 is required for the stability of the RAS protein in TNBC cell lines. Ubiquitination assays showed that RAS was polyubiquitinated by overexpression of WDR76 and exogenously expressed LRPPRC led to the increased ubiquitination of RAS (Figure S8G,H). Meanwhile, Western blotting showed that the expression of MYC protein in MDA‐MB‐231 and BT549 cells was increased after LRPPRC knockdown (Figures 5F and S8B). Collectively, these data indicated that LRPPRC knockdown reduces the stability of WDR76 mRNA via m^6^A modification, resulting in decreased WDR76 expression, decreased ubiquitination degradation of RAS protein, increased expression of RAS and MYC protein and enhanced glutamine‐dependent metabolism.

### 3.7 LRPPRC promotes TNBC progression and synthetic lethality with glutaminase inhibitors in xenograft models

**FIGURE 6:**
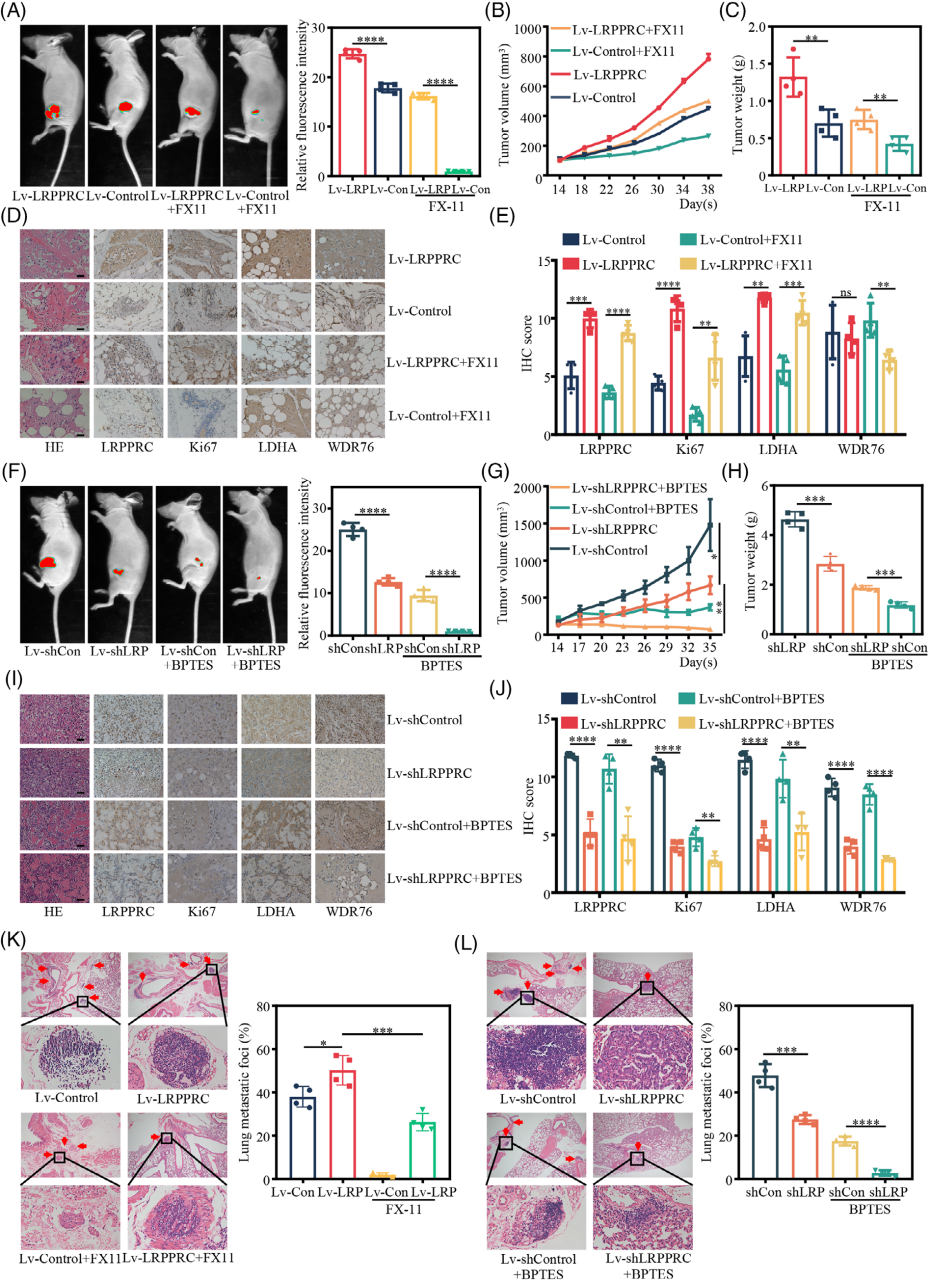
Leucine‐rich pentatricopeptide repeat containing protein (LRPPRC) promotes triple‐negative breast cancer (TNBC) progression and synthetic lethality with glutaminase inhibitors in vivo. (A) Representative bioluminescence images of mice after subcutaneous injection of LRPPRC overexpression or vector transfected BT549 cells treated with lactate dehydrogenase A (LDHA) inhibitor FX‐11 or solvent, respectively (left panel), and quantification of regional bioluminescence imaging results (right panel) (*n* = 4 for each group). (B) The tumours volume was monitored every 3 days, and tumours growth curves were generated. (C) The tumours were extracted and weighed. (D) Sections of tumours were stained with anti‐LRPPRC anti‐Ki‐67, anti‐LDHA and anti‐WD40 repeat domain‐containing protein 76 (WDR76) antibodies by immunohistochemical (IHC) staining. Scale bar, 20 μm. (E) Quantification of positive staining of LRPPRC, Ki‐67, LDHA and WDR76 in xenografted tumours. (F) Representative bioluminescence images of mice after subcutaneous injection of knockdown or vector transfected MDA‐MB‐231 cells treated with glutamine inhibitors BPTES or solvent, respectively (left panel), and quantification of regional bioluminescence imaging results (right panel) (*n* = 4 for each group). (G) The tumour volume was monitored every 3 days, and tumour growth curves were generated. (H) The tumours were extracted and weighed. (I) Sections of tumours were stained with anti‐LRPPRC anti‐Ki‐67, anti‐LDHA and anti‐WDR76 antibodies by IHC. Scale bar, 20 μm. (J) Quantification of positive staining of LRPPRC, Ki‐67, LDHA and WDR76 in xenografted tumours. (K) Representative haematoxylin–eosin (H&E) staining (left panel), quantification of lung metastatic colonisation (right panel) of nude mice (*n* = 4 for each group) treated with tail vein injection of LRPPRC overexpression or vector transfected BT549 cells treated with LDHA inhibitor FX‐11 or solvent, respectively. (L) Representative H&E staining (left panel), quantification of lung metastatic colonisation (right panel), of nude mice (*n* = 4 for each group) with tail vein injection of knockdown or vector transfected MDA‐MB‐231 cells treated with glutamine inhibitors BPTES or solvent, respectively. Values are the mean ± standard deviation (S.D.) of *n* = 3 independent experiments.

We developed a subcutaneous tumourigenesis model and a metastatic xenograft model to further confirm the oncogenic role of LRPPRC in vivo. First, LRPPRC‐overexpressing BT549 and control cells were injected subcutaneously into nude mice, respectively. After 7 weeks, the mice were sacrificed and the tumours were harvested (Figure S9A). Similarly, overexpression of LRPPRC resulted in an obvious increase in the growth and weight of subcutaneous xenograft tumours, and this effect was attenuated by FX‐11 (Figure 6A–C). IHC staining showed that the expression of Ki‐67 was increased, and this upregulation was diminished by FX‐11 (Figure 6D,E). LRPPRC knockdown MDA‐MB‐231 cells and control cells were subcutaneously administered into nude mice, respectively. After 7 weeks, the mice were sacrificed and the tumours were isolated (Figure S9B). In contrast, LRPPRC knockdown resulted in significantly reduced growth and weight of subcutaneous xenograft tumours. Moreover, LRPPRC knockdown plus BPTES, an inhibitor of glutaminase, accelerated this reduction (Figure 6F–H). IHC staining showed that the expression level of Ki‐67 and WDR76 was decreased in xenograft tumours induced by LRPPRC knockdown in MDA‐MB‐231 cells, while the inhibitor of glutaminase BPTES further lowered the level of Ki‐67 (Figure 6I,J).

Next, we utilised the metastatic xenograft model to examine the lung metastasis of TNBC cells 8 weeks after inoculation. The injection of LRPPRC‐overexpressing BT549 cells greatly increased the ability of TNBC cells to develop secondary tumours in the lung, and this effect was attenuated by FX‐11 (Figures 6K and S9C). In contrast, injection of LRPPRC knockdown MDA‐MB‐231 cells substantially impaired the ability of TNBC cells to form secondary tumours in the lung, and LRPPRC knockdown plus BPTES accelerated this reduction (Figures 6L and S9D). Collectively, these results indicated that LRPPRC promotes TNBC progression by upregulating LDHA expression, and the knockdown of LRPPRC in combination with the glutaminase inhibitor BPTES induced synthetic lethality in vivo.

### 3.8 FX‐11 plus BPTES induced synthetic lethality in LRPPRC‐positive TNBC PDX and patient‐derived organoid models

**FIGURE 7:**
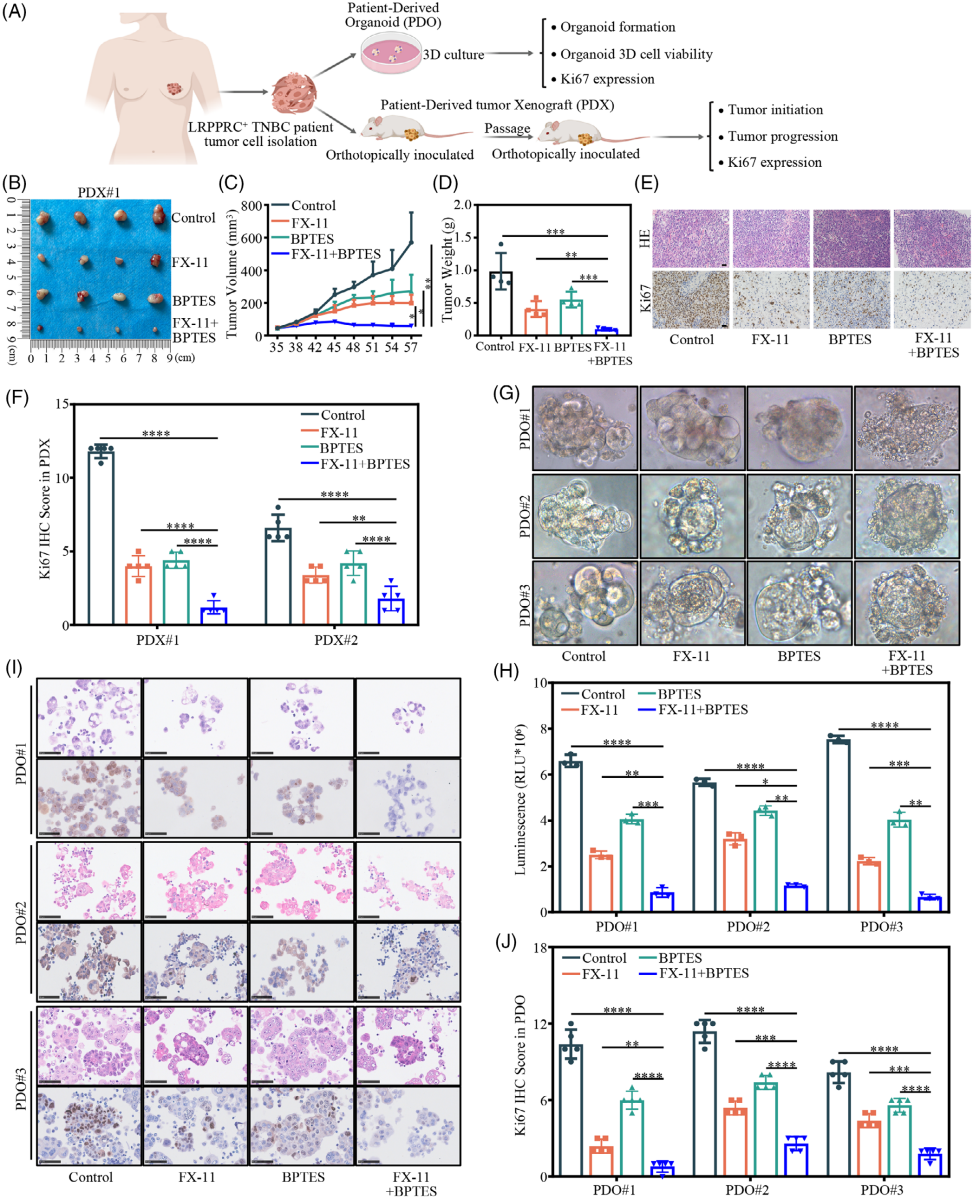
FX‐11 in combination with BPTES induced synthetic lethality in leucine‐rich pentatricopeptide repeat containing protein‐positive (LRPPRC^+^) triple‐negative breast cancer patient (TNBC)‐derived xenograft (PDX) and patient‐derived organoids (PDOs). (A) Graphical illustration of LRPPRC^+^ TNBC PDX and PDO mouse models. (B) The xenograft tumours of PDX mice treated with FX‐11, BPTES, FX‐11 combined with BPTES and vehicle control were collected. (C) Tumour volume was monitored in PDX mice every 3 days, and tumour growth curves were generated. (D) The tumours in PDX mice were extracted and weighed. (E) Sections of tumours in PDX mice were stained with anti‐Ki‐67 antibodies by immunohistochemical (IHC) staining. Scale bar, 20 μm. (F) Quantification of Ki‐67‐positive staining in PDX tumours. (G) Bright‐field images depicting the major phenotypes of TNBC organoids treated with FX‐11, BPTES, FX‐11 combined with BPTES and vehicle control, respectively. (H) Viability of organoids detected by CellTiter‐Glo 3D cell viability assay after the indicated treatments as described in (G). (I) Histological and IHC images showing the organisation structure and status of proliferation marker (Ki‐67) in organoids after the indicated treatments as described in (G). Scale bar, 50 μm. (J) Quantification of Ki‐67‐positive staining in organoids. Values are the mean ± standard deviation (S.D.) of *n* = 3 independent experiments.

To better mimic clinical status, we established preclinical TNBC models, PDX and patient‐derived organoid (PDO) models and treated them with FX‐11, BPTES or combination therapy, to further verify the synthetic lethality induced by LRPPRC knockdown plus glutaminase inhibitor in LRPPRC‐positive (LRPPRC^+^) TNBC (Figure 7A). First, we investigated the synergistic effect of the LDHA inhibitor FX‐11, a potent downstream target of LRPPRC, in combination with the glutaminase inhibitor BPTES in vitro. The colony formation assay showed that the clonogenic capacity of the combined treatment group was significantly inhibited compared to that of the models treated with FX‐11 or BPTES alone (Figure S10A–D). CCK‐8 assay showed that cell proliferation in the combined treatment group was significantly decreased (Figure S10E,F). Meanwhile, IHC staining showed that the expression patterns of the breast cancer markers ER, PR, HER2 and LRPPRC were well preserved in PDO/PDX models and parental tumour tissues (Figure S10G,H). The effect of the combination therapy was evaluated in the two LRPPRC^+^ PDX models. In line with the in vitro findings, the results revealed that FX‐11 combined with BPTES had the most significant suppressive effect on tumour growth (Figures 7B–D and S10I–K). IHC staining further demonstrated that Ki67 expression was significantly downregulated in the FX‐11 plus BPTES group (Figures 7E,F and S10L). Meanwhile, in the LRPPRC^+^ TNBC PDO models, combination therapy led to the most significant reduction in tumour organoid formation and cell viability (Figure 7G,H). Moreover, Ki67 immunostaining of PDOs showed a dramatic decrease in the FX‐11 plus BPTES group (Figure 7I,J). These findings suggested that targeted inhibition of the LRPPRC/LDHA pathway, in combination with glutaminase inhibitors, induced synthetic lethality in LRPPRC^+^ TNBC PDX and PDO models.

## 4 DISCUSSION

LRPPRC, a member of the PPR family that plays important roles in RNA stability, splicing and editing, has been shown to be dysregulated during tumour progression. Notably, LRPPRC has recently been identified as a reader for m^6^A modification sites. Nonetheless, the mechanism of LRPPRC as an m^6^A reader in TNBC remains poorly understood. In the present study, we found that LRPPRC was significantly upregulated in TNBC cells and could recognise m^6^A modification sites, suggesting that LRPPRC is implicated in the conversion of deregulated m^6^A modifications into pro‐tumourigenic signals. Meanwhile, using metabolomic methods, metabolite detection and cell phenotype assays, we demonstrated that LRPPRC enhanced glycolysis, thereby promoting tumour progression both in vivo and in vitro. Furthermore, using TNBC cell lines, LRPPRC‐knocked down xenograft mice, LRPPRC^+^ TNBC PDX and PDO models, we demonstrated that targeted knockdown of LRPPRC combined with a glutaminase inhibitor resulted in synthetic lethality in TNBC cells. Mechanistically, by integrating the m^6^A‐seq findings and transcriptome sequencing results, we found that LRPPRC directly binds to the m^6^A site on LDHA mRNA and maintains the stability of this mRNA to promote glycolysis. While LRPPRC knockdown reduced glycolysis, its role in promoting WDR76 mRNA stability through m^6^A modification pathway was weakened, then WDR76 decreased and its ubiquitination degradation of RAS decreased, RAS and MYC protein expression increased, and glutamine metabolism was enhanced. Our study revealed, for the first time, that LRPPRC‐mediated oncogenic m^6^A modification is involved in the metabolic reprogramming in TNBC, which may lead to a novel therapeutic strategy.

LRPPRC has been widely studied in various malignancies. It has been found to promote the progression of breast cancer, and its overexpression as an m^6^A RNA modulator is associated with poor prognosis of breast cancer.^22^ Consistent with previous studies, our study found that LRPPRC was significantly overexpressed in TNBC, and could recognise m^6^A modification sites, thereby promoting TNBC progression. In addition, multiple studies have demonstrated that LRPPRC plays an important role in energy metabolism, including oxidative phosphorylation and lipid metabolism.^23^ Interestingly, through metabolomic assay and cellular metabolic characterisation, we found that LRPPRC regulated glycolysis in TNBC cells, and knockdown of LRPPRC inhibited glycolysis. This further confirmed the specific role of LRPPRC in energy metabolism and the significance of the glycolytic process in highly proliferative TNBC cells.^24^

Previous studies have shown that LRPPRC plays an important role in the regulation of mRNA stability. For instance, LRPPRC forms a complex with SLIRP to suppress mRNA degradation,^25^ and lncRNA DANCR guides LRPPRC to stabilise the mRNA of Interleukin‐11 (IL‐11), Cyclin D1 (CCND1) and Plasminogen Activator, Urokinase (PLAU).^26^ Moreover, LRPPRC interacts with Beclin‐1 and Bcl‐2 to form a ternary complex that maintains the stability of Bcl‐2.^27^ Studies have shown that m^6^A modifications can either enhance or attenuate mRNA stability, depending on the localisation of the m^6^A reader protein in the cytoplasm.^28^ For example, the m^6^A reader protein YTHDF2 can mediate the degradation of m^6^A‐containing RNAs in mammalian cells,^29^, ^30^ and IGF2BPs selectively recognise m^6^A‐modified transcripts to promote mRNA stability.^31^, ^32^ Recent studies have shown that LRPPRC upregulates Programmed Death Ligand 1 (PD‐L1) expression by increasing the stability of PD‐L1 mRNA in an m^6^A‐dependent manner.^33^ In this regard, LRPPRC acts as a cytoplasmic m^6^A reader protein that also promotes the stability of LDHA and WDR76 mRNA in an m^6^A‐dependent manner. We further examined LDHA and identified the LRPPRC–m^6^A–LDHA axis as a critical molecular signalling pathway involved in LRPPRC‐mediated tumourigenesis and metastasis of TNBC. LDHA executes the final step of the Warburg effect as a catalyst that catalyses the reduction of pyruvate to lactate.^34^ In support of our data, LDHA has been reported to promote cancer proliferation, survival, invasion and metastasis by regulating cellular ATP levels, modulating cancer stem cell phenotypes and protecting the tumour from reactive oxygen species damage.^35^, ^36^, ^37^, ^38^ A recent study has also shown that m^6^A methylation mediates stability of the LDHA mRNA, which is regulated by the m^6^A reader protein IGF2BP2.^39^ Overall, our study identified LDHA as a novel downstream target of LRPPRC in an m^6^A‐dependent manner.

Recent studies have revealed that m^6^A RNA methylation is extensively involved in the metabolic reprogramming of tumour cells.^40^ Meanwhile, studies have shown that metabolic reprogramming is critical to cancer and that the Warburg effect is coupled with glutamine metabolism in cancer cells.^18^ Of note, our results showed that LRPPRC knockdown in TNBC cells attenuated cellular glycolysis, but, at the same time, enhanced cellular glutamine metabolism, which, in combination with a glutaminase inhibitor, induced synthetic lethality. This may be ascribed to the regulation of WDR76 mRNA stability by LRPPRC through the m^6^A pathway. In line with our results, previous studies have shown that inhibition of glycolysis promoted the virus life cycle in cancer cells treated with oncolytic viruses, but this promotion was largely dependent on exogenous glutamine.^41^ In addition, studies have shown that MYC stimulates mitochondrial glutaminolysis and leads to glutamine addiction.^21^ Meanwhile, RAS can enhance MYC protein stability,^20^ and RAS protein is regulated by WDR76 ubiquitination degradation.^19^ Our study further demonstrated that WDR76 regulated RAS protein degradation through ubiquitination, thereby mediating MYC protein expression in TNBC, and m^6^A methylation played an important role in metabolic reprogramming and its mediation of LRPPRC in the regulation of glycolysis and its metabolic coupling to glutamine in TNBC.

The PDX and PDO models share some major histological and genetic features with their donor tumours and remain stable across passages.^42^, ^43^ Two promising preclinical models have been used in biological studies for biomarker identification and the development of personalised drug strategies.^44^ We further confirmed that the inhibition of LRPPRC/LDHA signalling plus glutaminase inhibitors could induce synthetic lethality in LRPPRC^+^ TNBC PDX and PDO models. Our study provided a firm experimental basis for a new therapeutic approach that targets LRPPRC for the treatment of TNBC.

While our study found that synthetic lethality could be induced by the LDHA inhibitor FX‐11 in combination with the glutaminase inhibitor BPTES in LRPPRC^+^ TNBC cell line‐derived xenograft and preclinical models, it has yet to be determined whether this combination therapy is effective against TNBC with under‐expression of LRPPRC or non‐TNBC. Moreover, whether LRPPRC plays an essential role in luminal and HER2‐positive breast cancer warrants further investigation.

**FIGURE 8:**
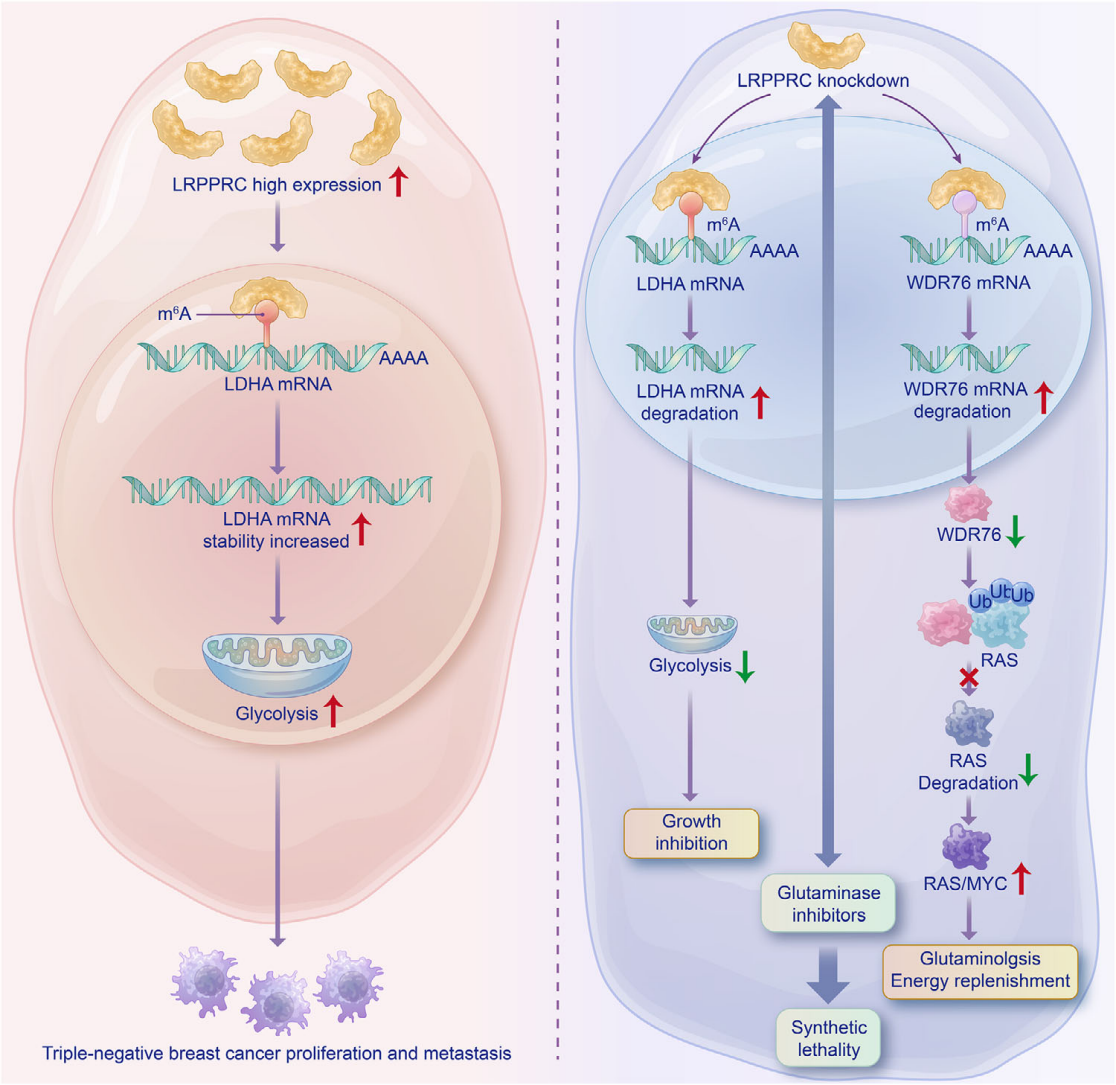
A graphical representation of leucine‐rich pentatricopeptide repeat containing protein (LRPPRC) promoting glycolysis and targeting LRPPRC in combination with glutaminase inhibitors inducing synthetic lethality in triple‐negative breast cancer (TNBC).

In conclusion, our study demonstrated that the m^6^A‐dependent LRPPRC–LDHA/WDR76 axis played a crucial role in TNBC progression. Notably, through the m^6^A pathway, LDHA‐mediated glycolysis was upregulated by LRPPRC and WDR76/RAS/MYC‐mediated glutamine metabolism was enhanced after LRPPRC knockdown in TNBC, indicating that m^6^A plays an important role in TNBC metabolic reprogramming, at least in part, by maintaining the stability of m^6^A‐containing oncogenic mRNAs (Figure 8). Collectively, our study provides a new strategy for the treatment of LRPPRC^+^ TNBC and may expand the application of drugs that target tumour metabolism in TNBC.

## AUTHOR CONTRIBUTIONS

Bo Zhang and Jianying Chen designed and supervised the experiments. Yuanhang Yu, Huifang Deng and Wenwen Wang conducted the experiments. Yuanhang Yu, Shihan Xiao, Renjing Zheng and Lianqiu Lv contributed reagents. Wenwen Wang and Han Wang provided clinical samples. Yuanhang Yu and Huifang Deng wrote the manuscript.

## CONFLICT OF INTEREST STATEMENT

The authors declare they have no conflicts of interest.

## ETHICS STATEMENT

The experimental mice were housed in the specific pathogen‐free facility of the Huazhong University of Science and Technology, and all procedures were performed in strict accordance with the guidelines of the Institutional Animal Care and Use Committee, Huazhong University of Science and Technology (S2815), Wuhan, China. Human tumour tissues were obtained from Wuhan Union Hospital, Huazhong University of Science and Technology, Wuhan, China. The study was approved by the Institutional Human Ethics Committee of Tongji Medical College, Huazhong University of Science and Technology (S093), Wuhan, China. Informed consent was obtained from all tissue donors.

## Supporting information

Supporting information

## Data Availability

All data are available in the main text or the Supporting Information.
