# Supplementary material for: LRPPRC promotes glycolysis by stabilising LDHA mRNA and its knockdown plus glutamine inhibitor induces synthetic lethality via m6A modification in triple‐negative breast cancer
Source: Clin Transl Med. 2024 Feb 19;14(2):e1583. doi: 10.1002/ctm2.1583 (PMC10875709; doi:10.1002/ctm2.1583)
Supplement: Supplementary file 1 — Supporting information [file CTM2-14-e1583-s001.docx]

Supplementary Materials for

**LRPPRC promotes glycolysis by stabilizing LDHA mRNA and its knockdown plus glutamine inhibitor induces synthetic lethality**

**via m6A modification in triple-negative breast cancer**

Figs. S1 to S10

Tables S1 to S2


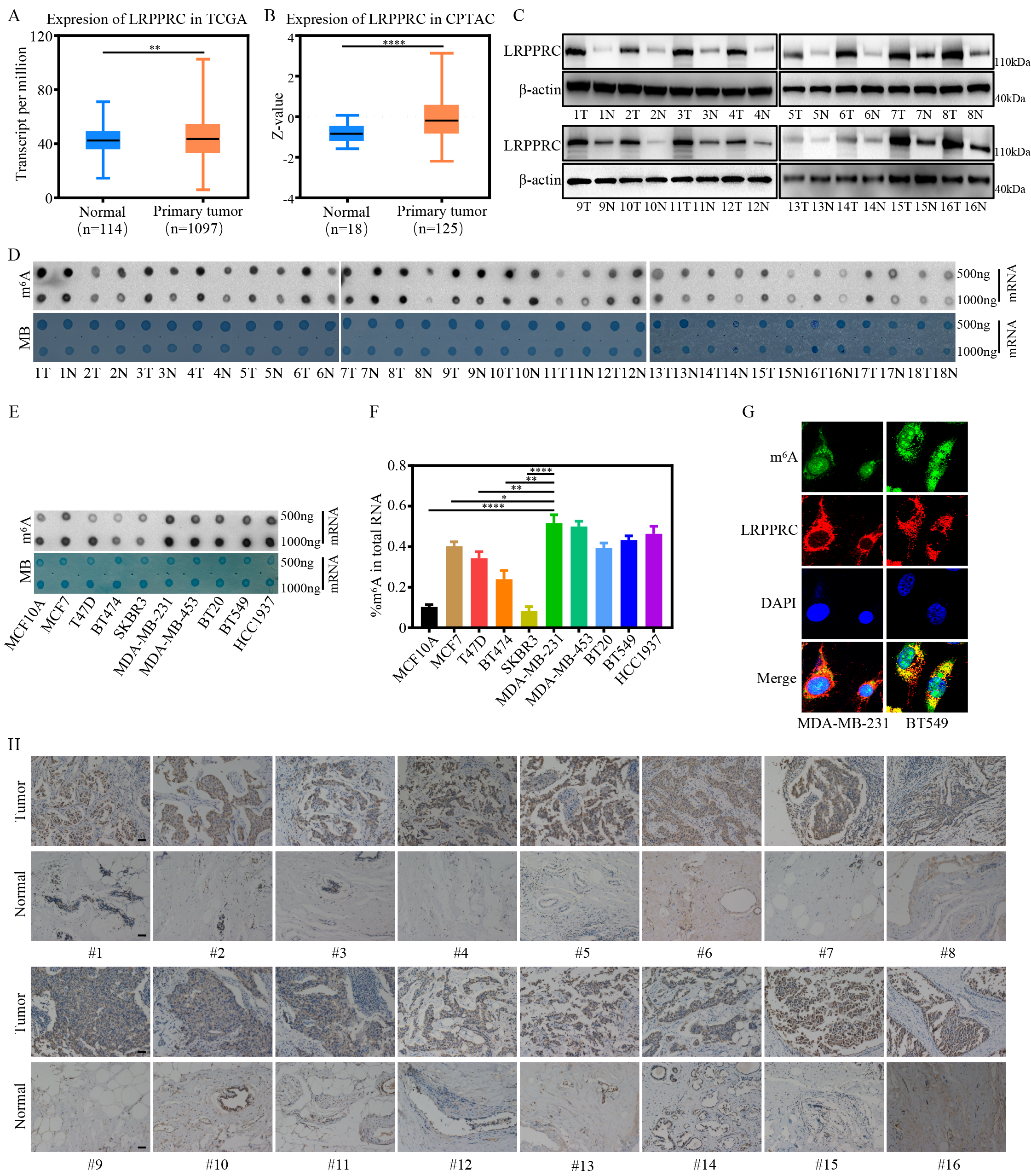


**Figure. S1. LRPPRC and m^6^A modification levels were up-regulated in triple negative breast cancer.** (A-B) Relative mRNA and protein expression levels of LRPPRC in breast cancer based on TCGA and CPTAC datasets, respectively. (C) Expression of LRPPRC in 16 pairs of TNBC tissues and adjacent normal tissues, as detected by Western blot analysis. (D) The mRNAs isolated from 18 pairs of TNBC tissues and adjacent normal tissues as shown by dot blot analyses with an anti-m^6^A antibody, and MB (methylene blue) staining serving as the loading control. (E) The mRNAs isolated from normal breast cell line and breast cancer cell lines were used in dot blot analyses, with an anti-m^6^A antibody, and MB (methylene blue) staining served as the loading control. (F) The m^6^A RNA levels in the normal breast cell line and breast cancer cell lines were colorimetrically detected by using an m^6^A RNA methylation quantification kit. (G) Cellular localization of LRPPRC and m6A modification sites in TNBC cells as demonstrated by immunofluorescence staining using LRPPRC and m^6^A antibody, respectively. (H) Representative IHC images of LRPPRC in 16 pairs of TNBC tissues and adjacent normal tissues. Scale bar, 20 μm. Western blot images are representative of three independent experiments. Values are the mean ±  s.d. of n= 3 independent experiments.


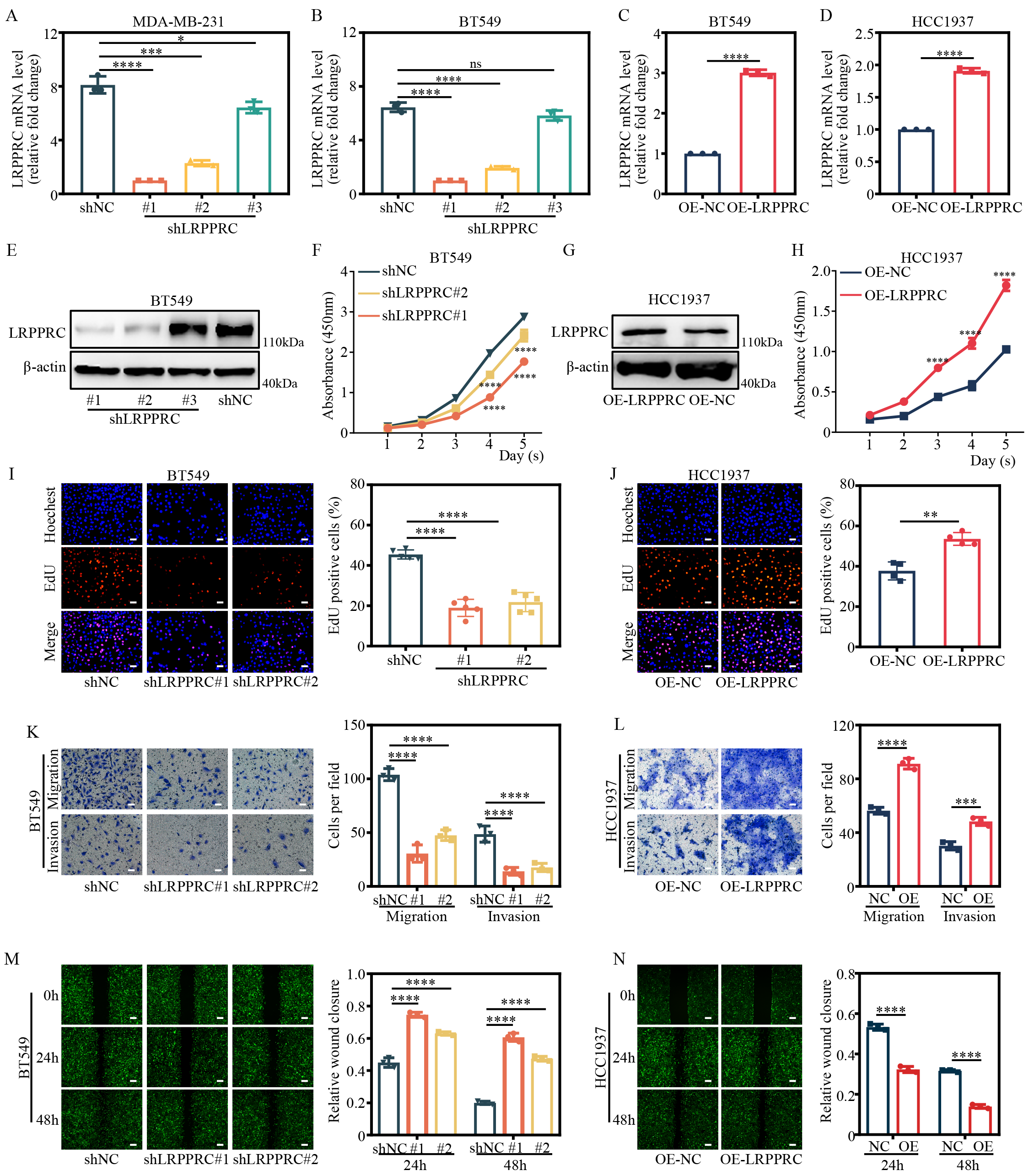


**Figure. S2. LRPPRC promoted the proliferation, migration and invasion of TNBC cells *in vitro*.** (A, B) mRNA expression of LRPPRC upon LRPPRC knockdown in MDA-MB-231 and BT549 cell lines as revealed by qRT-PCR. (C, D) Elevated mRNA expression of LRPPRC upon LRPPRC overexpression in BT549 and HCC1937 cell lines as revealed by qRT-PCR. (E) Decreased LRPPRC expression in BT549 transfected with LRPPRC-inhibiting shRNAs as displayed by Western blotting. (F) Cell growth after LRPPRC knockdown in BT549 cells as shown by CCK8 assays. (G) Elevated LRPPRC expression in HCC1937 transfected with plasmids overexpressing LRPPRC as exhibited by western blot analysis. (H) Cell growth after LRPPRC overexpression in HCC1937 cells as shown by CCK8 assays. (I, J) LRPPRC knockdown BT549 and LRPPRC overexpression HCC1937 cells, respectively, as shown by EdU assays. Representative images are shown in the left panel, Scale bar, 50 μm, and Quantification of EdU positive cells are shown in the right panel. (K, L) Effects of LRPPRC on migration and invasive abilities of LRPPRC-knocked-down BT549 and LRPPRC-overexpressing HCC1937 cells as determined by transwell invasion assays, Scale bar, 50 μm. (M, N) Effects of LRPPRC on migration abilities of LRPPRC-knocked-down BT549 and LRPPRC-overexpressing HCC1937 cells as determined by wound healing assays, Scale bar, 50 μm.


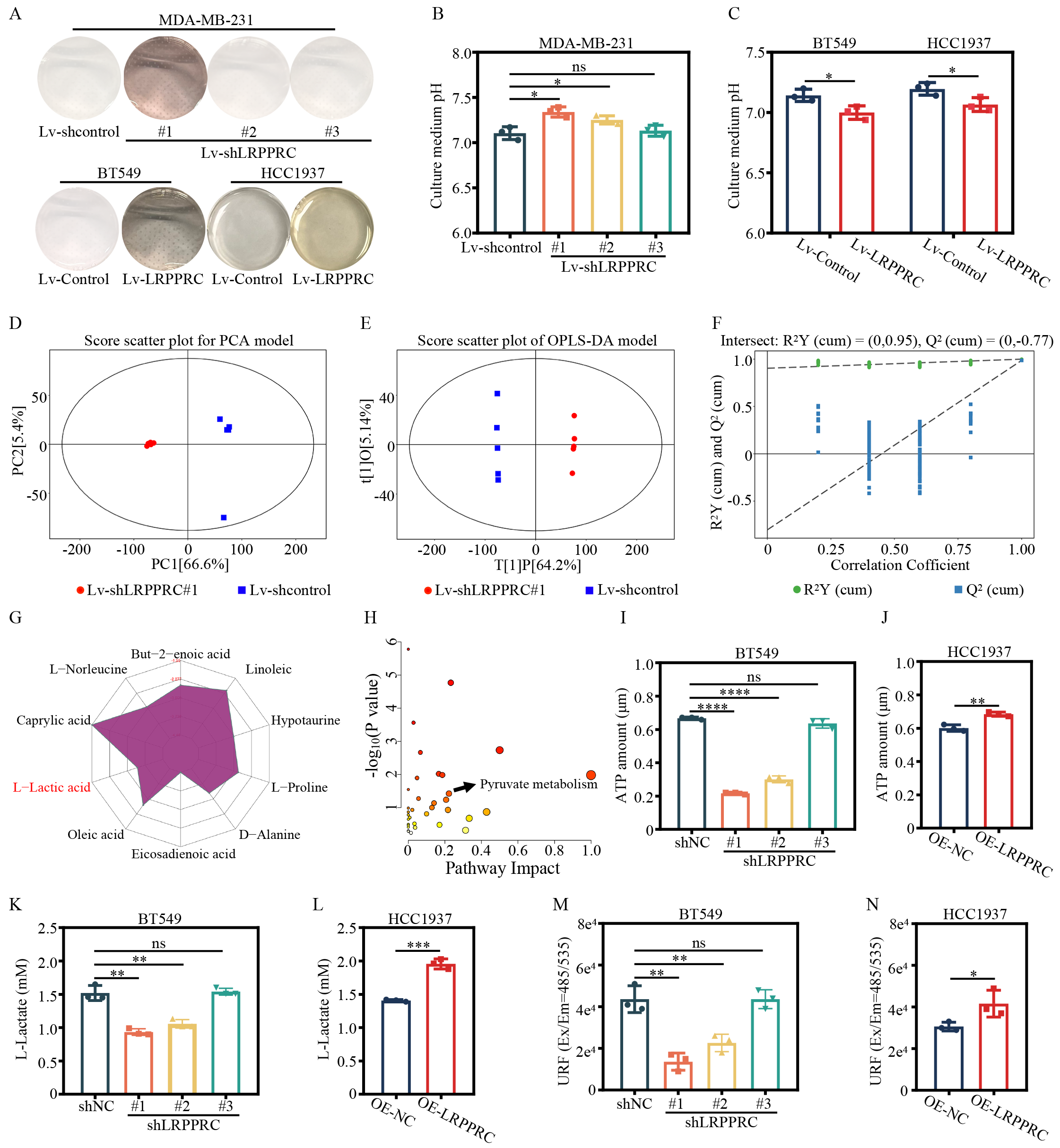


**Figure. S3. LRPPRC promoted glycolysis in triple negative breast cancer.** (A) Representative images of colors of the indicated MDA-MB-231, BT549 and HCC1937 cells culture medium. (B, C) The pH value of the indicated MDA-MB-231, BT549 and HCC1937 culture medium. (D) Score scatter plot of PCA model for control and LRPPRC-knocked-down MDA-MB-231 cells. X axis and Y axis represent the contributions of persons to the first two principal components (PC1 and PC2). Each scatter represents a sample, and scatter shapes and colors indicate different experimental groupings. (E) Score scatter plot of OPLS-DA model for control and LRPPRC-knocked-down MDA-MB-231 cells. X axis and Y axis represent the contributions of persons to the first two principal components (t[1]P and t[1]O). Each scatter represents a sample, and scatter shapes and colors denote different experimental groupings. (F) Permutation test of OPLS-DA model for control and LRPPRC-knocked-down MDA-MB-231 cells. (G) Radar chart analysis for control and knockdown LRPPRC MDA-MB-231 cells. Each grid line represents a difference fold, and the purple shade is composed of the line of difference fold for each substance. (H) KEGG enrichment analysis was used to identify the differential metabolites between control and LRPPRC-knocked-down MDA-MB-231 cells. (I, J) Intracellular ATP levels in LRPPRC-knocked-down BT549 (I) and LRPPRC-overexpressing HCC1937 (J) cells, respectively. (K, L) Glycolysis cell-based assays of LRPPRC-knocked-down BT549 (K) and LRPPRC-overexpressing HCC1937 (L) cells, respectively. L-lactate was detected as the end product of glycolysis. (M, N) Glucose uptake cell-based assays of LRPPRC-knocked-down BT549 (M) and LRPPRC-overexpressing HCC1937 (N) cells, respectively. The experiments were replicated 3 times, and the data are expressed as Mean ±  s.d.


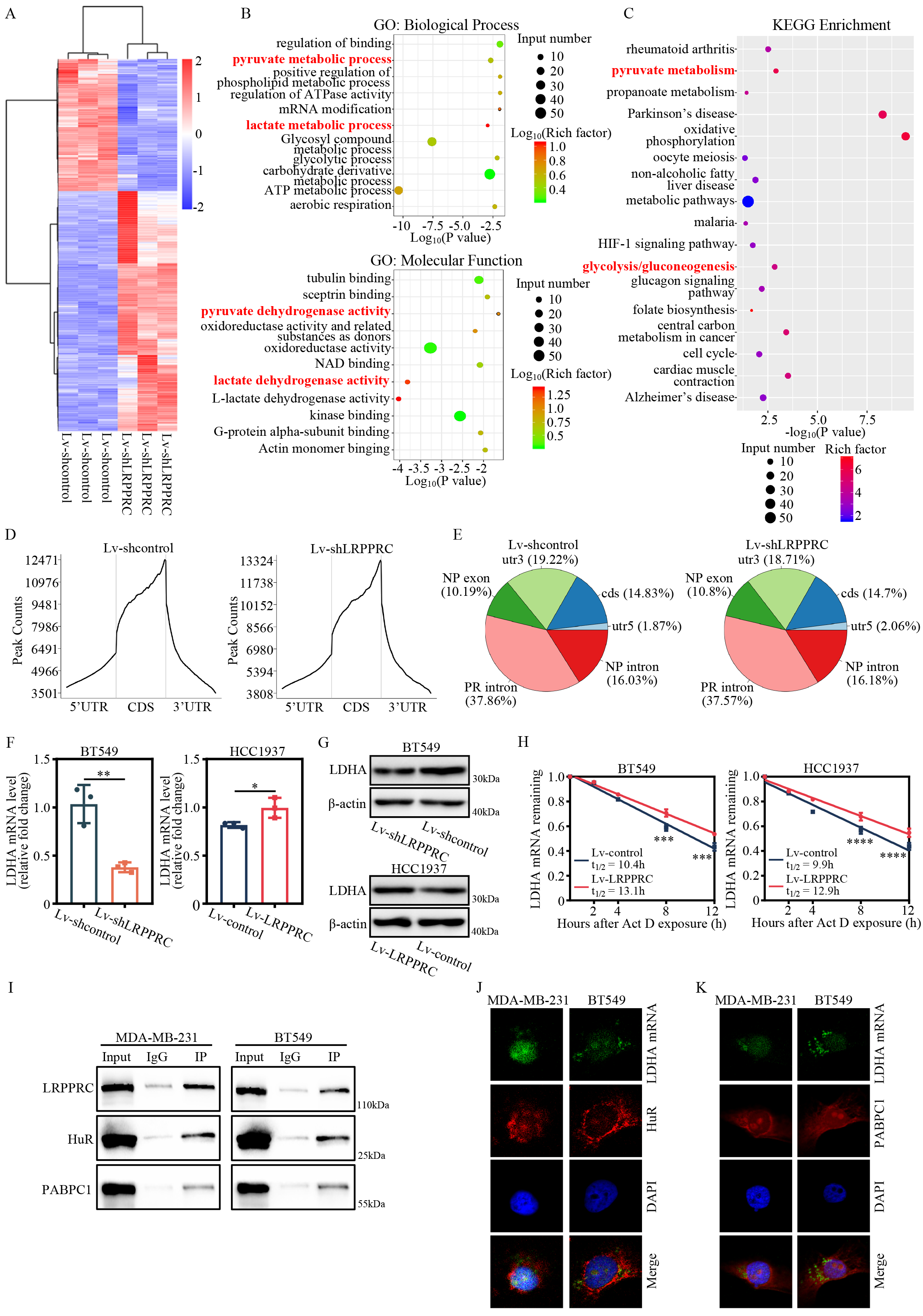


**Figure. S4. The analysis identified LDHA as a target of LRPPRC in TNBC.** (A) Differentially-expressed genes identified by RNA-seq in Heatmap. (B) GO enrichment analysis of differentially-expressed genes identified by RNA-seq. (C) Enrichment of KEGG pathways for significantly down-regulated genes in LRPPRC-knocked-down and control MDA-MB-231 cells. (D) The density of m^6^A peaks in 3´non-overlapping transcript segments: 5´ untranslated region (UTR), coding sequence (CDS), and 3´UTR. (E) The distribution of m^6^A peaks in different RNA subgroups. (F) The mRNA expression of LDHA upon LRPPRC knockdown in BT549 and LRPPRC overexpression in HCC1937 cells, respectively, as revealed by qRT-PCR. (G) LDHA expression upon LRPPRC knockdown in BT549 and LRPPRC overexpression in HCC1937 cells, respectively, as displayed by western blotting. (H) The levels of LDHA expression in LRPPRC overexpression and control TNBC cells treated with actinomycin D (5 µg/mL) at the indicated time points were detected by qRT-PCR. (I) Co-IP and Western blot assays revealing the interaction between endogenous LRPPRC with HuR and PABPC1 in MDA-MB-231 and BT549 cells. (J, K) Fluorescence in situ hybridization of LDHA mRNA and fluorescence immunostaining of HuR (J) or PABPC1 (K) in MDA-MB-231 and BT549 cells. Images are representative of three independent experiments. Western blot images are representative of three independent experiments. Values are the mean ±  s.d. of n= 3 independent experiments.


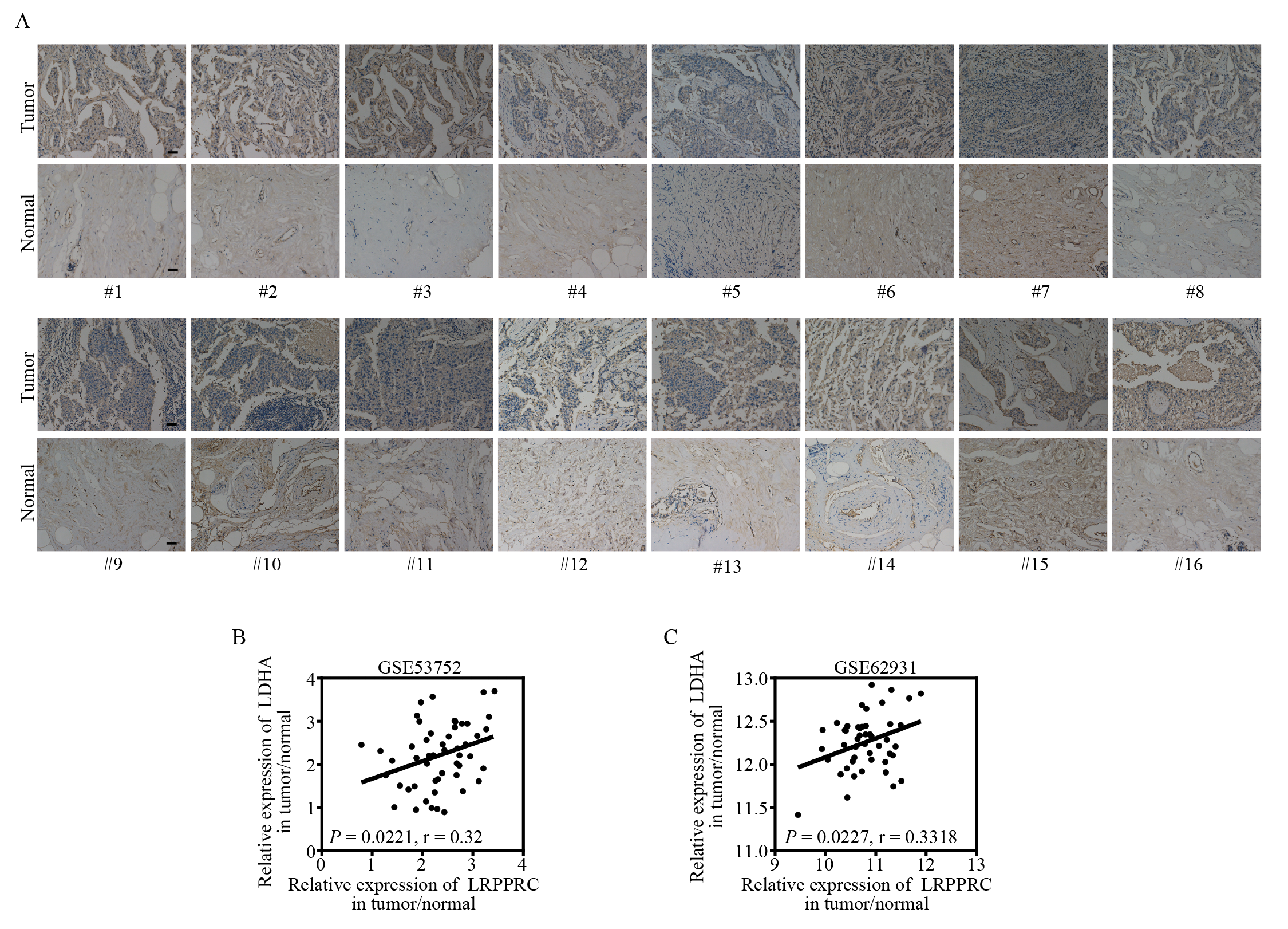


**Figure. S5. LDHA was overexpressed in TNBC.** (A) Representative IHC images of LRPPRC in 16 pairs of TNBC tissues and adjacent normal tissues. Scale bar, 20 μm. (B, C) The correlation between LRPPRC and LDHA in the GEO TNBC databases (Pearson’s correlation).


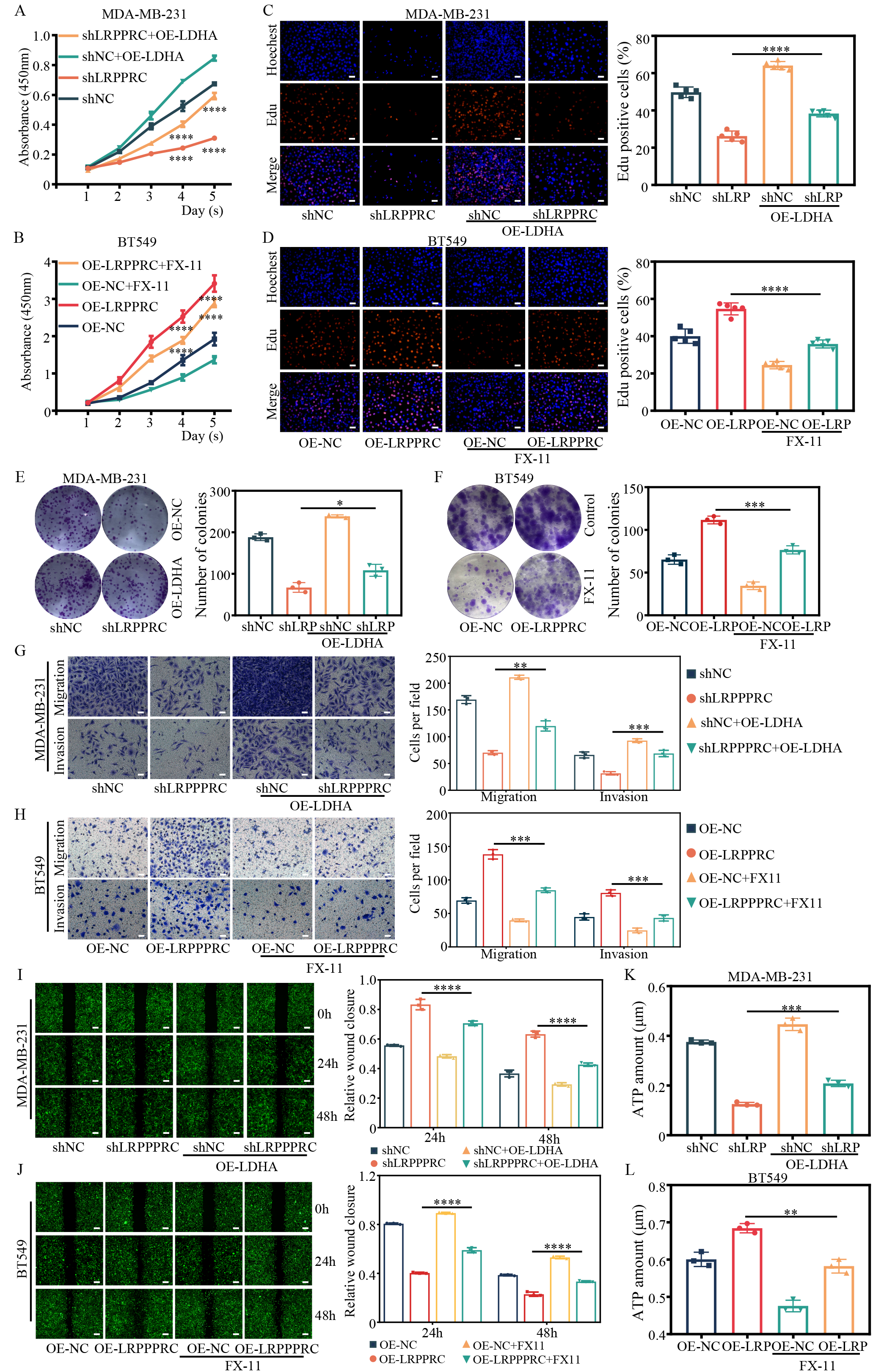


**Figure. S6. LRPPRC promoted TNBC progression via LDHA.** (A, B) Cell growth after LRPPRC knockdown in MDA-MB-231 cells transfected with plasmids overexpressing LDHA or empty vector control (A) and LRPPRC-overexpressing BT549 cells treated with FX-11 or DMSO (B), respectively, as shown by CCK8 assays. (C, D) Cell growth after LRPPRC knockdown in MDA-MB-231 cells transfected with plasmids overexpressing LDHA or empty vector control (C) and LRPPRC-overexpressing BT549 cells treated with FX-11 or DMSO (D), respectively, as shown by EdU assays, Scale bar, 50 μm. (E, F) Cell growth after LRPPRC knockdown in MDA-MB-231 cells transfected with plasmids overexpressing LDHA or empty vector control (E) and LRPPRC-overexpressing BT549 cells treated with FX-11 or DMSO (F), respectively, as determined by colony formation assays. (G, H) Effects of LRPPRC on migration and invasive ability of LRPPRC-knocked-down MDA-MB-231 cells transfected with plasmids overexpressing LDHA or empty vector control (G) and LRPPRC-overexpressing BT549 cells treated with FX-11 or DMSO (H), respectively, as determined by transwell invasion assays, Scale bar, 50 μm. (I, J) Effects of LRPPRC on migration ability of LRPPRC-knocked-down MDA-MB-231 cells transfected with LDHA constructs or empty vector control (I) and LRPPRC-overexpressing BT549 cells treated with FX-11 or DMSO (J), respectively, as determined by wound healing assay, Scale bar, 50 μm. (K, L) Intracellular ATP levels in LRPPRC-knocked-down MDA-MB-231 cells transfected with plasmids overexpressing LDHA or empty vector control (K) and LRPPRC-overexpressing BT549 cells treatmented with FX-11 or DMSO (L), respectively. Values are the mean ±  s.d. of n= 3 independent experiments.


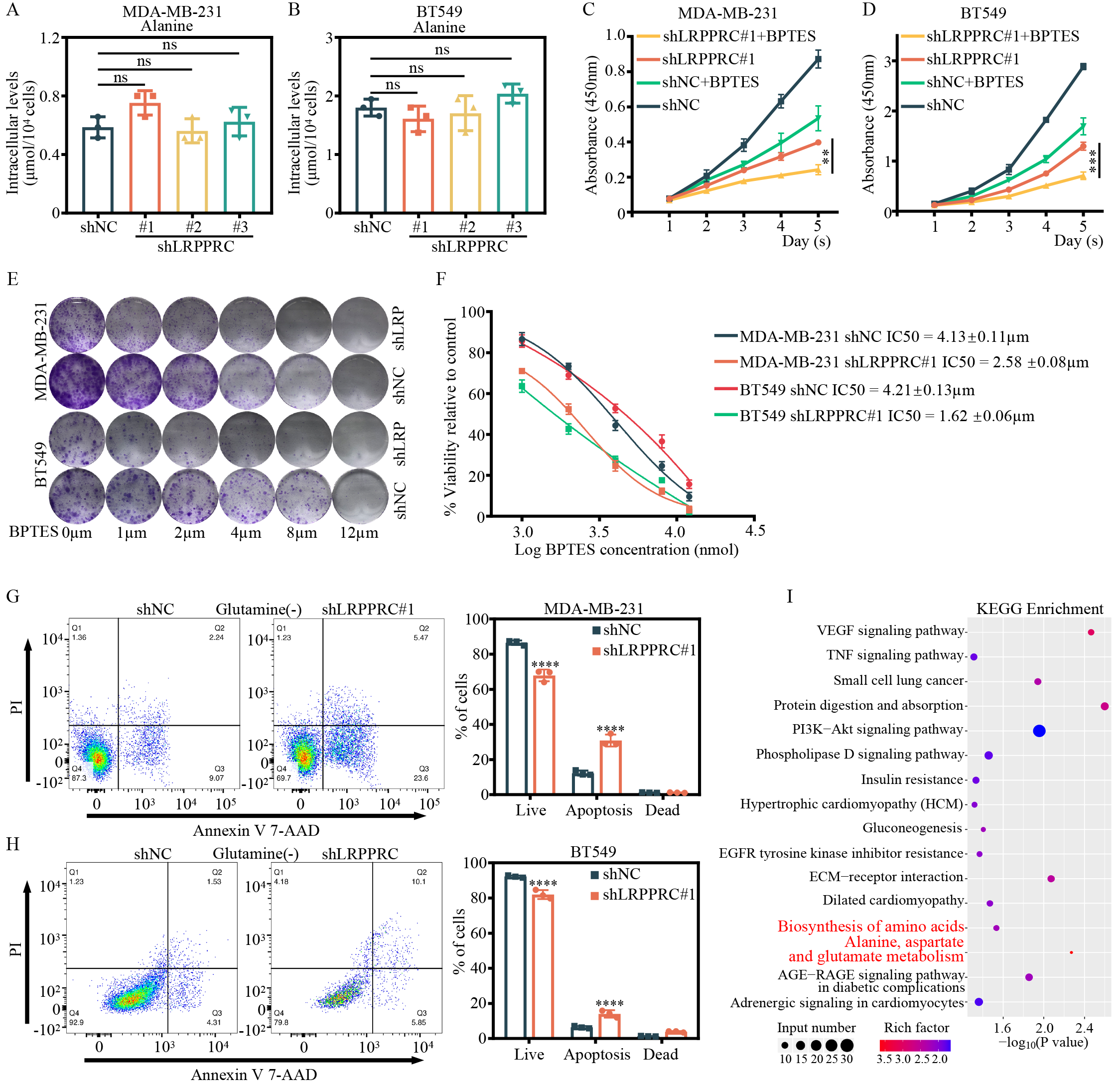


**Figure. S7. Knockdown of LRPPRC induced synthetic lethality with glutamine inhibitors BPTES.** (A, B) Intracellular alanine levels in LRPPRC-knocked-down MDA-MB-231 and BT549 cells, respectively. (C, D) Cell growth after LRPPRC knockdown in MDA-MB-231 and BT549 cells and control cells treated with BPTES or DMSO, respectively, as shown by CCK8 assays. (E) Cell growth after LRPPRC knockdown in MDA-MB-231 and BT549 cells and control cells treated with BPTES or DMSO, respectively, as determined by colony formation assays. (F) Quantification of cell clones described in (E). (G, H) LRPPRC knocked-down MDA-MB-231 and BT549 cells and control cells treated with BPTES or DMSO, respectively. Apoptosis analysis was performed, and representative flow cytometrical images are shown (left panel); Quantification of apoptotic cell percentage is shown (right panel). (I) Enrichment of KEGG pathway genes significantly upregulated in LRPPRC knocked-down MDA-MB-231 cells. Values are the mean ± s.d. of n= 3 independent experiments.


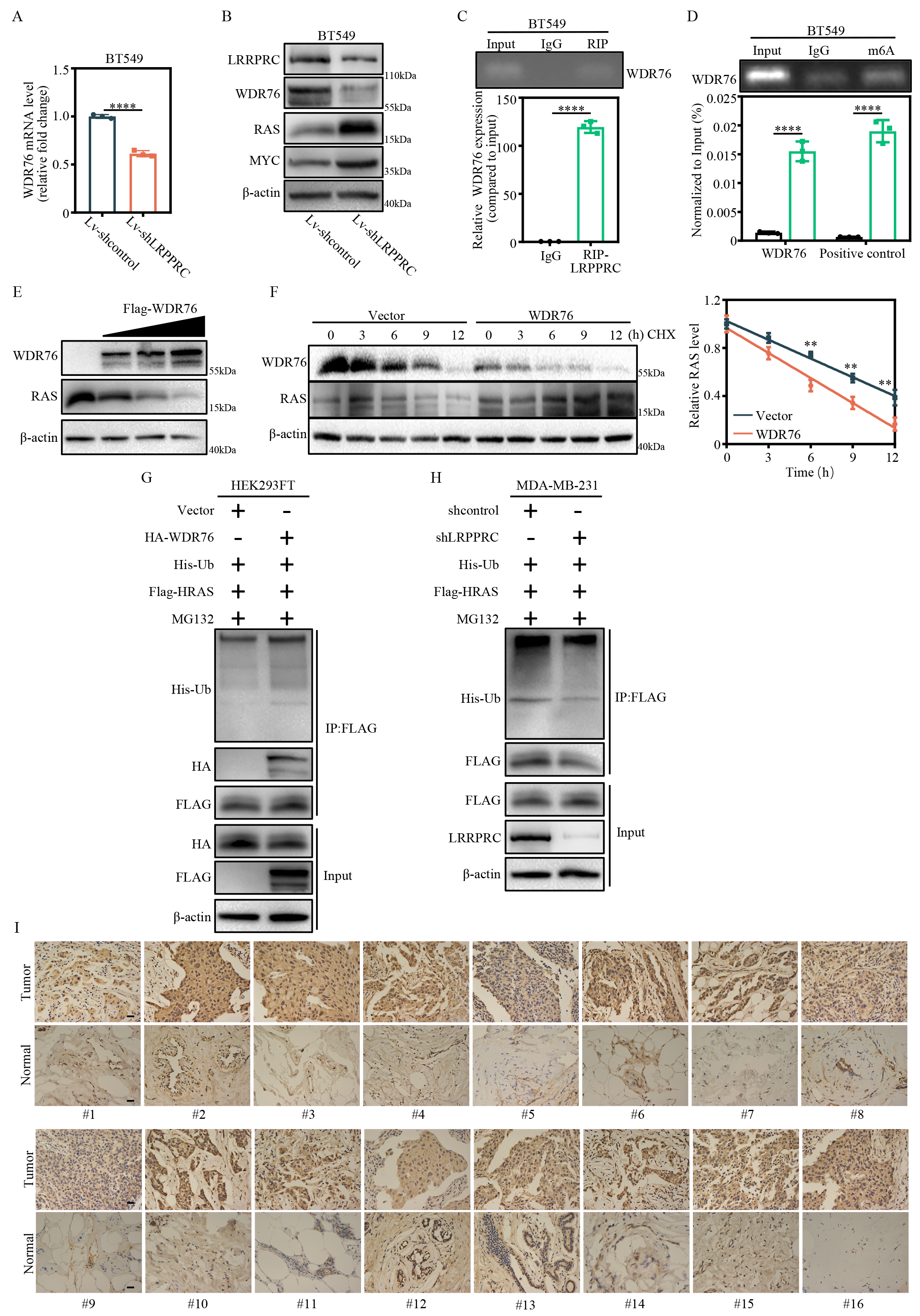


**Figure. S8. LRPPRC regulates RAS/MYC protein expression through WDR76-mediated ubiquitination degradation of RAS**. (A) The mRNA expression of WDR76 upon LRPPRC knockdown in MDA-MB-231 by qRT-PCR. (B) WDR76, RAS and MYC expression upon LRPPRC knockdown in MDA-MB-231 by western blotting. (C) RIP-PCR validating LRPPRC binding to GPT2 mRNA in MDA-MB-231. (D) Gene-specific m^6^A qPCR validation of m^6^A levels on GPT2 mRNA in MDA-MB-231. (E) 293T cells were transfected with dose-dependent expression of Flag-WDR76, cells were immunoblotted with the indicated antibodies. (F) WDR76 overexpression or control MDA-MB-231 cells were treated with 20μM cycloheximide (CHX; protein synthesis inhibitor) for the indicated times. Cells were immunoblotted with anti-WDR76 and anti-RAS antibodies. (G, H) Western blotting analysis of the ubiquitination level of RAS derived from Co-IP in 293T (G) and MDA-MB-231 (H) cells transfected with the indicated constructs, respectively. (I) Representative IHC images of WDR76 in 16 pairs of TNBC tissues and adjacent normal tissues. Scale bar, 20 μm.


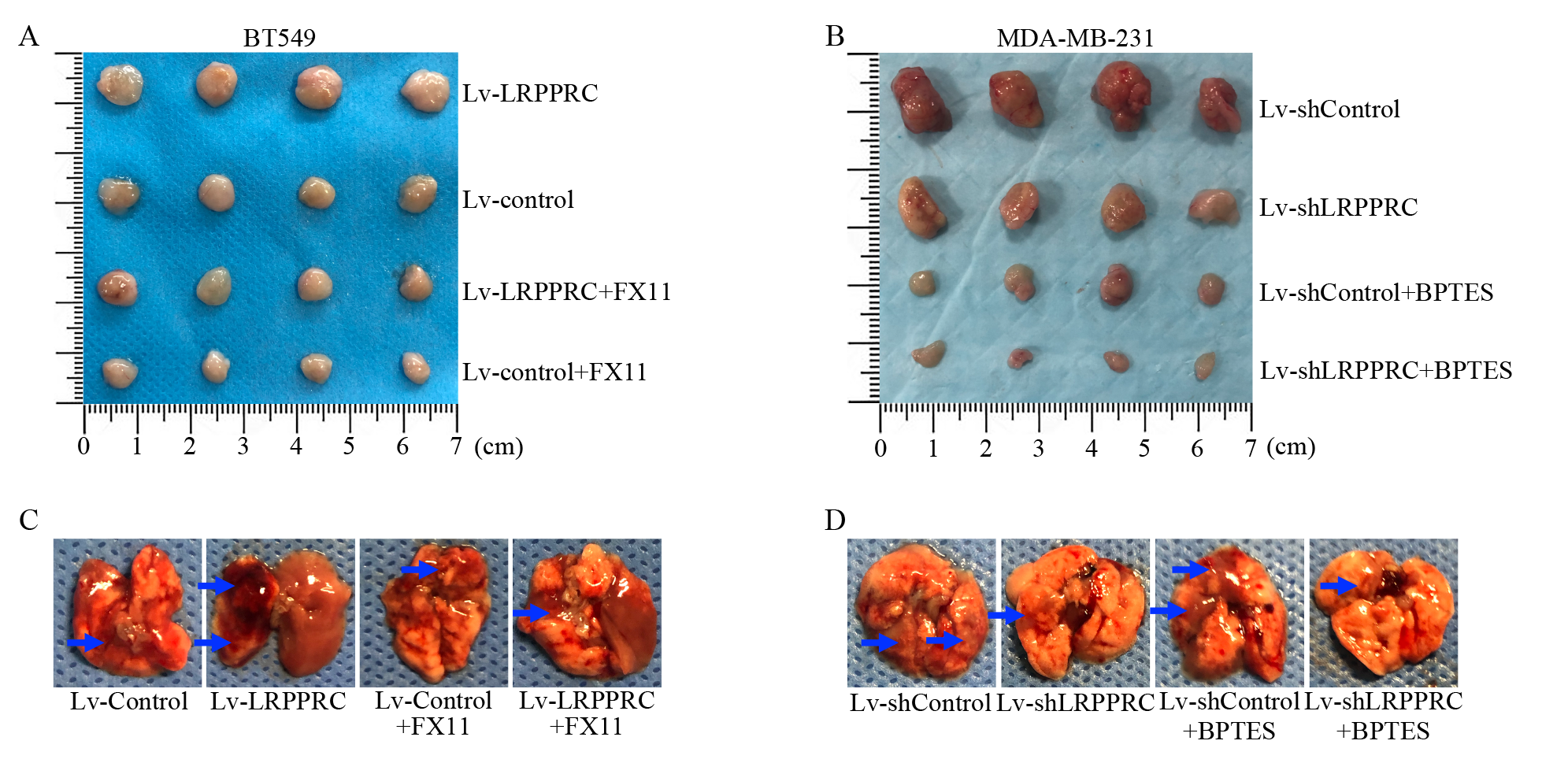


**Figure. S9. LRPPRC promotes TNBC progression *in vivo*.** (A) A representative image of the tumor xenografts harvested 38 days after the subcutaneous injection of 5*10^6^ LRPPRC overexpression or vector transfected BT549 cells into the nude mouse treated with LDHA inhibitor FX-11 (2 mg/kg, i.p., daily, for 3 weeks) or solvent, respectively. (B) A representative image of the tumor xenografts taken 38 days after the subcutaneous injection of 5*10^6^ knockdown or vector transfected MDA-MB-231 cells into the nude mouse treated with LDHA inhibitor FX-11 (12.5 mg/kg, i.p., daily, for 2 weeks) or solvent, respectively. (C) Lung tissues of nude mice were treated with the LDHA inhibitor FX-11 (2 mg/kg, i.p., daily for 3 weeks) or solvent by tail vein injection of LRPPRC overexpression or vector transfected BT549 cells, respectively. (D) Lung tissues of nude mice were treated with glutamine inhibitors BPTES (12.5 mg/kg, i.p., daily, for 2 weeks) or solvent by tail vein injection of knockdown or vector transfected MDA-MB-231 cells, respectively.


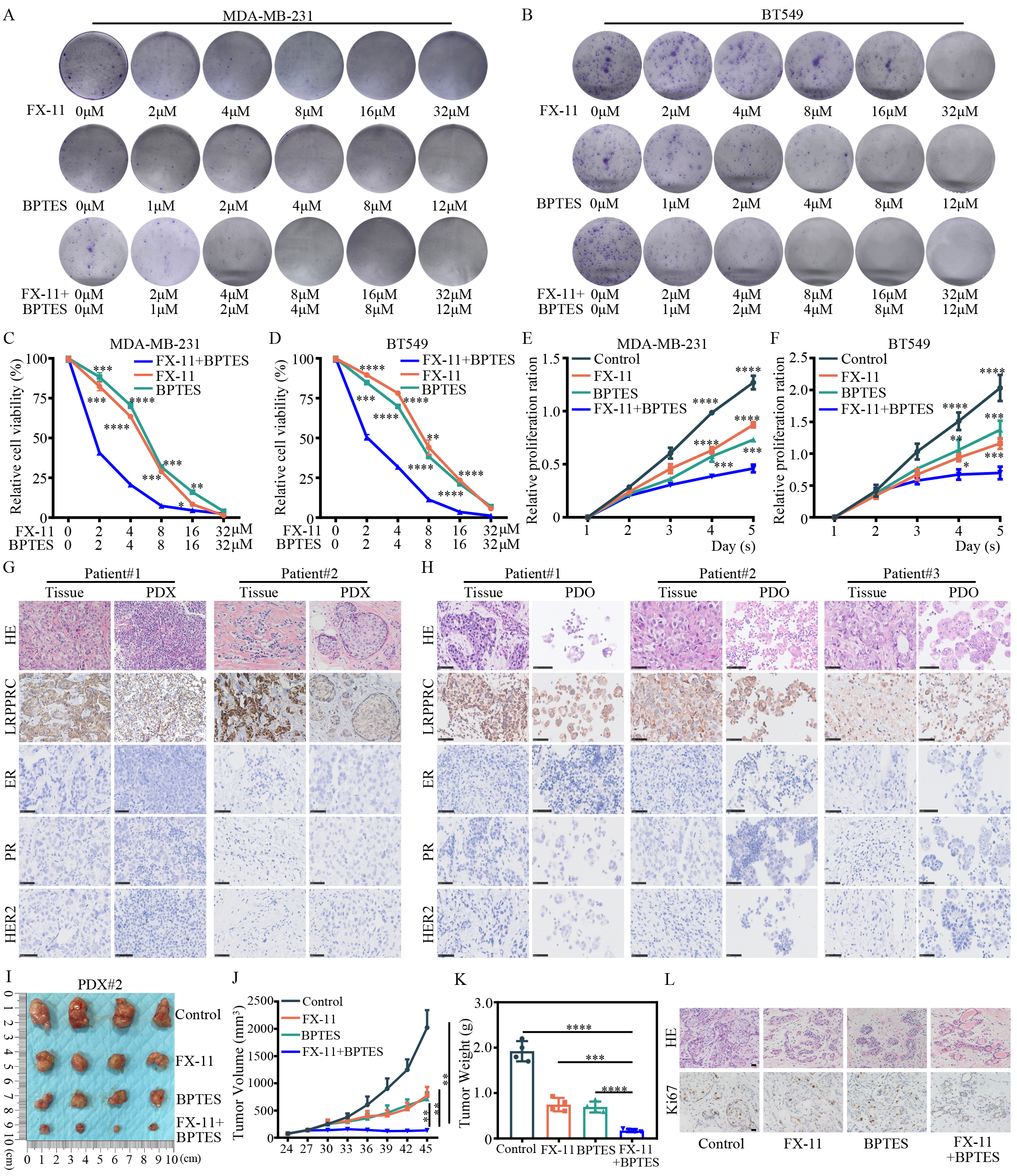


**Figure. S10. FX-11 plus BPTES induced synthetic lethality in LRPPRC-positive triple negative breast cancer.** (A, B) Colony formation assays on MDA-MB-231 (A) and BT549 (B) cells treated with 0, 2, 4, 8, 16 or 32 μM FX-11; 0, 1, 2, 4, 8 or 12 μM BPTES or both of them, respectively. (C, D) Cell viability assays on MDA-MB-231 (C) and BT549 (D) cells treated with 0, 2, 4, 8, 16 or 32 μM FX-11; 0, 1, 2, 4, 8 or 12 μM BPTES or both of them, respectively. (E, F) CCK8 assays were performed to determine the cell growth of MDA-MB-231 (E) and BT549 (F) cells treated with 5 μM FX-11, 5 μM BPTES, or both of them or vehicle control, respectively. (G) Histological and immunohistochemical images showing the status of LRPPRC, Ki-67 and breast cancer-related markers (ER, PR, and HER2) in primary tumors and PDXs, Scale bar: 50 μm. (H) Histological and immunohistochemical images showing the status of LRPPRC, Ki-67 and breast cancer-related markers (ER, PR, and HER2) in primary tumors and organoids. Scale bar: 50 μm. (I) The xenograft tumors of PDX mice treated with FX-11, BPTES, FX-11 combined with BPTES and vehicle control were collected. (J) Tumor volume was monitored in PDX mice every three day, and tumor growth curves were generated. (K) The tumors in PDX mice were extracted and weighed. (L) Sections of tumors in PDX mice were stained with anti-Ki-67 antibodies by IHC, Scale bar: 20 μm.

**Supplementary Tables.**

**Supplementary Table S1. The sequences of sh-LRPPRCs.**

| **NO.** | **Accession** | **Target Seq** | **CDS** | **GC%** |
| --- | --- | --- | --- | --- |
| sh-LRPPRC#1 | NM_133259 | ccTCAAAGGAATGCAAGAATT | 59..4243 | 31.58% |
| sh-LRPPRC#1 | NM_133259 | cgCAGCTTTAAGAGGTGAAAT | 59..4243 | 36.84% |
| sh-LRPPRC#1 | NM_133259 | ccTCGCATTATTGAATGCATA | 59..4243 | 31.58% |
| Description | Homo sapiens leucine rich pentatricopeptide repeat containing(LRPPRC), mRNA. | | | |

| **Supplementary Table S2. Primers used in this study.** | |
| --- | --- |
| Primers used for qRCR (5’-3’) and RIP-qPCR | |
| LRPPRC forward | AGATGGCCCAAGTGTCTTTG |
| LRPPRC reverse | AAGCACAATGGAGGGTGAAC |
| LDHA forward | GGATGAGCTTGCCCTTGTTGA |
| LDHA reverse | GACCAGCTTGGAGTTCGCAGTTA |
| WDR76 forward | AGCTACAACCCAAGAGAACGG |
| WDR76 reverse | CCCGAAAAATCCAGGGATGGT |
| GAPDH forward | GTCACCAGGGCTGCTTTTAACTC |
| GAPDH reverse | CAGCATCGCCCCACTTGATTTTG |
|  |  |
| Primers used for m^6^A real-time RCR (5’-3’) | |
| LDHA-MeRIP forward | ATGATGTCTTCCTTAGTGTT |
| LDHA-MeRIP reverse | CTCAGAAGTCAGAGTCACCT |
| WDR76-MeRIP forward | ACAACACTTTCACTGGGCGA |
| WDR76-MeRIP reverse | GCATGGCATTGATGGAACAC |
| EEF1A1 Positve forward | CGGTCTCAGAACTGTTTGTTTC |
| EEF1A1 Positve reverse | AAACCAAAGTGGTCCACAAA |
| EEF1A1 Negative forward | GGATGGAAAGTCACCCGTAAG |
| EEF1A1 Negative reverse | TTGTCAGTTGGACGAGTTGG |
